# Quantum computational advantage with a programmable photonic processor

**DOI:** 10.1038/s41586-022-04725-x

**Published:** 2022-06-01

**Authors:** Lars S. Madsen, Fabian Laudenbach, Mohsen Falamarzi. Askarani, Fabien Rortais, Trevor Vincent, Jacob F. F. Bulmer, Filippo M. Miatto, Leonhard Neuhaus, Lukas G. Helt, Matthew J. Collins, Adriana E. Lita, Thomas Gerrits, Sae Woo Nam, Varun D. Vaidya, Matteo Menotti, Ish Dhand, Zachary Vernon, Nicolás Quesada, Jonathan Lavoie

**Affiliations:** 1 Xanadu, Toronto, ON Canada; 2 National Institute of Standards and Technology, Boulder, CO USA

**Keywords:** Quantum simulation, Quantum optics, Quantum information, Information theory and computation, Single photons and quantum effects

## Abstract

A quantum computer attains computational advantage when outperforming the best classical computers running the best-known algorithms on well-defined tasks. No photonic machine offering programmability over all its quantum gates has demonstrated quantum computational advantage: previous machines^1,2^ were largely restricted to static gate sequences. Earlier photonic demonstrations were also vulnerable to spoofing^3^, in which classical heuristics produce samples, without direct simulation, lying closer to the ideal distribution than do samples from the quantum hardware. Here we report quantum computational advantage using Borealis, a photonic processor offering dynamic programmability on all gates implemented. We carry out Gaussian boson sampling^4^ (GBS) on 216 squeezed modes entangled with three-dimensional connectivity^5^, using a time-multiplexed and photon-number-resolving architecture. On average, it would take more than 9,000 years for the best available algorithms and supercomputers to produce, using exact methods, a single sample from the programmed distribution, whereas Borealis requires only 36 μs. This runtime advantage is over 50 million times as extreme as that reported from earlier photonic machines. Ours constitutes a very large GBS experiment, registering events with up to 219 photons and a mean photon number of 125. This work is a critical milestone on the path to a practical quantum computer, validating key technological features of photonics as a platform for this goal.

## Abstract (web summary)

Gaussian boson sampling is performed on 216 squeezed modes entangled with three-dimensional connectivity^5^, using Borealis, registering events with up to 219 photons and a mean photon number of 125.

## Main

Only a handful of experiments have used quantum devices to carry out computational tasks that are outside the reach of present-day classical computers^1,2,6,7^. In all of these, the computational task involved sampling from probability distributions that are widely believed to be exponentially hard to simulate using classical computation. One such demonstration relied on a 53-qubit programmable superconducting processor^6^, whereas another used a non-programmable photonic platform implementing Gaussian boson sampling (GBS) with 50 squeezed states fed into a static random 100-mode interferometer^1^. Both were shortly followed by larger versions, respectively enjoying more qubits^7,8^ and increased control over brightness and a limited set of circuit parameters^2^. In these examples, comparison of the duration of the quantum sampling experiment to the estimated runtime and scaling of the best-known classical algorithms placed their respective platforms within the regime of quantum computational advantage.

The superconducting quantum supremacy demonstrations serve as crucial milestones on the path to full-scale quantum computation. On the other hand, the choice of technologies used in the photonic machines^1,2^, and their consequential lack of programmability and scalability, places them outside any current proposed roadmap for fault-tolerant photonic quantum computing^9–11^ or any GBS application^12–18^. A demonstration of photonic quantum computational advantage incorporating hardware capabilities required for the platform to progress along the road to fault-tolerance is still lacking.

In photonics, time-domain multiplexing offers a comparatively hardware-efficient^19^ path for building fault-tolerant quantum computers, but also near-term subuniversal machines showing quantum computational advantage. By encoding quantum information in sequential pulses of light—effectively multiplexing a small number of optical channels to process information on a large number of modes^20^—large and highly entangled states can be processed with a relatively small number of optical components. This decouples the required component count and physical extent of the machine from the size of the quantum circuit being executed; provided device imperfections can be maintained sufficiently small, this decoupling represents a substantial advantage for scaling. Moreover, the relatively modest number of optical pathways and control components avoids many of the challenges of traditional, planar two-dimensional implementations of optical interferometers, which suffer from high complexity and burdensome parallel control requirements, especially when long-range connectivity is desired. Although attractive for scaling, hardware efficiency must not come at the cost of unnacceptably large errors. Implementations of time-domain multiplexing must therefore be tested in demanding contexts to validate their promise for building practically useful quantum computers.

Using time-domain multiplexing, large one- and two-dimensional cluster states have been deterministically generated^21–23^ with programmable linear operations implemented by projective measurements^24,25^, whereas similar operations have been implemented in ref. ^26^ using a single loop with reconfigurable phase. These demonstrations leverage low-loss optical fibre for delay lines, which allows photonic quantum information to be effectively buffered. Although groundbreaking, these demonstrations have remained well outside the domain of quantum computational advantage, as they lacked non-Gaussian elements and were unable to synthesize states of sufficient complexity to evade efficient classical simulation^27^. The demonstration of a set of hardware capabilities needed for universal fault-tolerant quantum computing, in the demanding context of quantum computational advantage, would serve as a validating signal that the corresponding technologies are advancing as needed. Yet no such demonstration is available for time-domain multiplexing.

In this work, we solve technological hurdles associated with time-domain multiplexing, fast electro-optical switching, high-speed photon-number-resolving detection technology and non-classical light generation, to build a scalable and programmable Gaussian boson sampler, which we name Borealis. These features allow us to synthesize a 216-mode state with a three-dimensional entanglement topology. This is particularly notable because three-dimensional cluster states are sufficient for measurement-based fault-tolerant quantum computing^28,29^; although the states we synthesize are themselves not cluster states, the device can be readily programmed to generate cluster states by selecting appropriate phase and beam-splitting ratios at the loops. Borealis uses 216 independent quantum systems to achieve quantum computational advantage, placing it well beyond the capabilities of current state-of-the-art classical simulation algorithms^30^. Our use of photon-number-resolving detectors unlocks access to sampling events with much larger total photon number, a regime inaccessible to earlier experiments that used traditional threshold detectors. In the same vein, our use of time-domain multiplexing allows us access to more squeezed modes without increasing the physical extent or complexity of the system. In addition, its output cannot be efficiently spoofed in cross-entropy benchmarks using a generalization of the most recent polynomial-time algorithms^3^. We leave as an open question to the community whether better polynomial-time algorithms for spoofing can be developed.

## Experiment

**Fig. 1:**
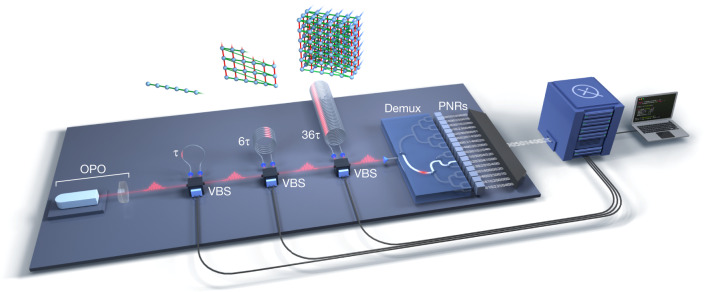
High-dimensional GBS from a fully programmable photonic processor. A periodic pulse train of single-mode squeezed states from a pulsed OPO enters a sequence of three dynamically programmable loop-based interferometers. Each loop contains a VBS, including a programmable phase shifter, and an optical fibre delay line. At the output of the interferometer, the Gaussian state is sent to a 1-to-16 binary switch tree (demux), which partially demultiplexes the output before readout by PNRs. The resulting detected sequence of 216 photon numbers, in approximately 36 μs, comprises one sample. The fibre delays and accompanying beamsplitters and phase shifters implement gates between both temporally adjacent and distant modes, enabling high-dimensional connectivity in the quantum circuit. Above each loop stage is depicted a lattice representation of the multipartite entangled Gaussian state being progressively synthesized. The first stage (*τ*) effects two-mode programmable gates (green edges) between nearest-neighbour modes in one dimension, whereas the second (6 *τ*) and third (36 *τ*) mediate couplings between modes separated by six and 36 time bins in the second and third dimensions (red and blue edges, respectively). Each run of the device involves the specification of 1,296 real parameters, corresponding to the sequence of settings for all VBS units.

The optical circuit we implement, depicted in Fig. 1, is fully programmable, provides long-range coupling between different modes and allows all such couplings to be dynamically programmed. It implements linear-optical transformations on a train of input squeezed-light pulses, using a sequence of three variable beamsplitters (VBSs) and phase-stabilized fibre loops that act as effective buffer memory for light, allowing interference between modes that are either temporally adjacent, or separated by six or 36 time bins. This system synthesizes a programmable multimode entangled Gaussian state in a 6 MHz pulse train, which is then partially demultiplexed to 16 output channels and sampled from using photon-number-resolving detectors.

Unlike some quantum algorithms whose correct functioning on a quantum computer can be readily verified using a classical computer, it remains an open question how to verify that a GBS device is operating correctly. In what follows, we present evidence that our machine is operating correctly, that is, it samples from the GBS distribution specified by the device transfer matrix *T* and vector of squeezing parameters **r**, which together define the ground truth of the experiment. In previous experiments^1,2^ the results were benchmarked against a ground truth obtained from tomographic measurements of a static interferometer, whereas for Borealis, the ground truth is obtained from the quantum program specified by the user, that is the squeezing parameters and phases sent to the VBS components in the device.

The transfer matrix is obtained by combining the three layers of VBSs acting over the different modes, together with common (to all modes) losses due to propagation and the finite escape efficiency of the source, as well as imperfect transmittance through the demultiplexing and detection systems; it corresponds classically (quantum mechanically) to the linear transformation connecting input and output electric fields (annihilation operators).

As noted in refs. ^5,31^, if one were to target a universal and programmable interferometer, with depth equal to the number of modes, that covers densely the set of unitary matrices, the exponential accumulation of loss would prohibit showing a quantum advantage. There are then two ways around this no-go result: one can either give up programmability and build an ultralow loss fixed static interferometer, as implemented in refs. ^1,2^, or give up universality while maintaining a high degree of multimode entanglement using long-ranged gates.

**Fig. 2:**
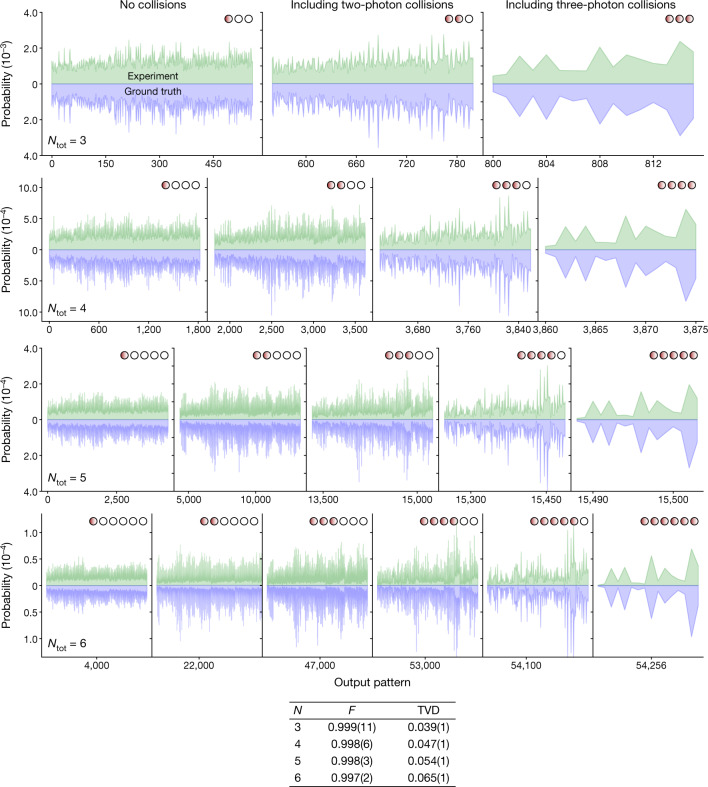
Experimental validation of the GBS device. Each panel compares experimentally obtained sample probabilities, against those calculated from the ground truth (*r*, *T*), for up to six-photon events in a 16-mode state. A total of 84.1 × 10^6^ samples were collected and divided according to their total photon number *N* and further split according to the collision pattern, from no collision (no more than one photon detected per PNR) to collisions of different densities (more than one photon per PNR). The overall fidelity (*F*) and TVD to simulations for each photon-number event is shown below. Further analysis of TVD for classical adversaries in the 16-mode GBS instance can be found in the Supplementary Information.

We first consider the regime of few modes and low photon number, in which it is possible to collect enough samples to estimate outcome probabilities, and also calculate these from the experimentally characterized lossy transmission matrix *T* and the experimentally obtained squeezing parameters **r** programmed into the device. In Fig. 2 we show the probabilities inferred from the random samples collected in the experiment and compare them against the probabilities for different samples *S* obtained from simulations, under the ground truth assumption. We cover the output pattern of all possible permutations $$(\begin{array}{c}N+M-1\\ N\end{array})$$, in which *N* is the number of photons, from 3 to 6, and *M* = 16 is the number of modes. To quantify the performance of Borealis we calculate the fidelity (*F*) and total variation distance (TVD) of the 3, 4, 5 and 6 total photon-number probabilities relative to the ground truth. For a particular total photon number, fidelity and TVD are, respectively, defined as $F={\sum }_{i}\sqrt{{p}_{i}{q}_{i}}$ (also known as the Bhattacharyya coefficient) and ${\rm{TVD}}={\sum }_{i}|{p}_{i}-{q}_{i}|/2$. Parameters *p*_*i*_ and *q*_*i*_ represent the theoretical and experimental probability of the *i*th output pattern, respectively, and are normalized by the probability of the respective total photon number. For the total photon-number sectors considered we find fidelities in excess of 99% and TVDs below or equal to 6.5%, thus showing that our machine is reasonably close to the ground truth in the low-*N* regime addressed by these data. Note that, because we are calculating all the possible probabilities with *N* photons, estimating outcome probabilities from the experimentally characterized transmission matrix would require us to obtain orders of magnitude more samples, beyond our current processing abilities. This limitation will lead to TVD growing as *N* increases and, beside the impractical computational cost, is the reason that data past *N* > 6 were left for subsequent benchmarks.

In an intermediate mode- and photon-number regime, we calculate the cross entropy of the samples generated by the experiment for each total photon-number sector for a high-dimensional GBS instance with *M* = 216 computational modes and total mean photon number $\bar{N}=21.120\pm 0.006$. For a set of *K* samples ${\{{S}_{i}\}}_{i=1}^{K}$, each having a total of *N* photons, the cross-entropy benchmark under the ground truth given by (**r**, *T*) is

1 $${\rm{XE}}({\{{S}_{i}\}}_{i=1}^{K})=\frac{1}{K}\mathop{\sum }\limits_{i=1}^{K}\mathrm{ln}\left(\frac{{{\rm{\Pr }}}^{(0)}({S}_{i})}{{\mathscr{N}}}\right),$$

where $${\mathscr{N}}={{\rm{\Pr }}}^{(0)}(N)/(\begin{array}{c}N+M-1\\ N\end{array})$$ is a normalization constant determined by the total number of ways in which *N* photons can be placed in *M* modes and Pr^(0)^(*N*) is the probability of obtaining a total of *N* photons under the ground truth assumption.

**Fig. 3:**
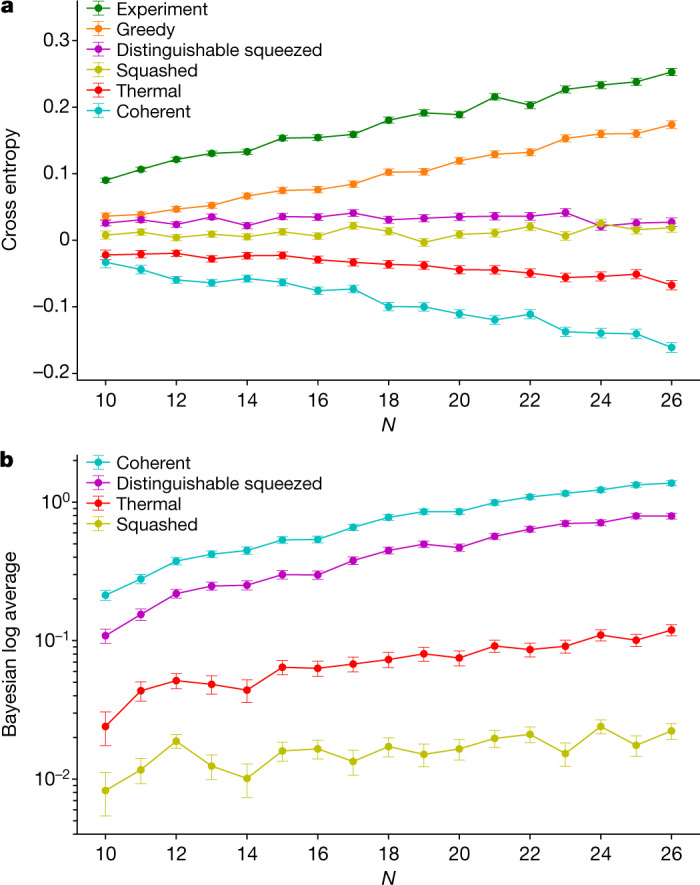
Benchmarks against the ground truth. **a**, Cross-entropy benchmark against the ground truth. Experimental samples from a high-dimensional GBS instance of 216 modes, averaging $\bar{N}=21.120\pm 0.006$ photons per sample, are bundled according to their total photon number *N*, from 10 to 26. Each point (score) corresponds to an average (equation (1)) over 10,000 samples per *N*. Genuine samples from the quantum hardware score higher than all classical spoofers, validating the high device fidelity with the ground truth. Error bars are standard errors of the mean. **b**, Bayesian log average score against the ground truth. Experimental samples from a 72-mode GBS instance and $\bar{N}=22.416\pm 0.006$ photon number per sample. Each score is averaged over 2,000 samples with *N* from 10 to 26. Error bars are standard errors of the mean. All scores are above zero, including error bar, indicating that the samples generated by Borealis are closer to the ground truth than from the adversarial distribution corresponding to squashed, thermal, coherent and distinguishable squeezed spoofers.

We then compare the average score (Fig. 3a) of the 10^6^ samples, divided in 10,000 samples per total photon number *N*, generated by our machine in the cross entropy against classical adversarial spoofers that try to mimic the ground truth distribution (**r**, *T*). These adversaries are constructed with the extra constraint that they must have the same first-order (mean) photon-number cumulants as the ground truth distribution. The five adversaries considered send (1) squashed, (2) thermal, (3) coherent and (4) distinguishable squeezed light into the interferometer specified by *T*, or (5) use a greedy algorithm to mimic the one- and two-mode marginal distributions of the ground truth, as was used in ref. ^3^ to spoof earlier large GBS experiments^1,2^. Squashed states (1) are the classical-Gaussian states with the highest fidelity to lossy-squeezed states^31^, that is they are optimal within the family of Gaussian states that are classical, and thus provide a more powerful adversary than thermal, coherent or distinguishable squeezed states, which were the only adversaries considered in previous photonic quantum computational advantage claims^1,2^. In all cases, the samples from Borealis perform significantly better than any adversary at having a high cross entropy with respect to the ground truth; equivalently, none of the adversaries are successful spoofers in this benchmark. In particular, the best-performing adversary—the greedy sampler—remains significantly below the experiment in cross-entropy, and shows no trend towards outperforming the experiment for larger *N*. Given the supercomputing resources and time needed to estimate all scores for *N* = 26 (22 h), we can extrapolate this time and estimate that it would take roughly 20 days to benchmark our data for *N* = 30. For this reason, and the lack of evidence that the scores may change in favour of any alternative to the ground truth, we are confident that the studied range of *N* = [10,26] is sufficient to rule out all classical spoofers considered, even in the regime in which it is unfeasible to perform these benchmarks.

Next, we consider another test—a Bayesian method similar to that used in other GBS demonstrations^1,2^. For each subset of samples generated in the experiment with a given total photon number *N*, we calculate the ratio of the probability that a sample *S* could have come from the lossy ground truth specified by *T* and **r** to the probability that *S* came from any of the alternative spoofing hypotheses (1)–(4). For a particular sample *S*_*i*_ and a particular adversary *I* this ratio is given by

2 $${R}^{0|I}({S}_{i})=\frac{{{\rm{\Pr }}}^{(0)}({S}_{i}|N)}{{{\rm{\Pr }}}^{(I)}({S}_{i}|N)}=\frac{{{\rm{\Pr }}}^{(0)}({S}_{i})\,{{\rm{\Pr }}}^{(I)}(N)}{{{\rm{\Pr }}}^{(I)}({S}_{i})\,{{\rm{\Pr }}}^{(0)}(N)}.$$

which allows us to form the Bayesian log average

3 $$\Delta {H}_{0|I}=\frac{1}{K}\mathop{\sum }\limits_{i=1}^{K}\mathrm{ln}\,{R}^{0|I}({S}_{i}).$$

If $\Delta {H}_{0|I} > 0$ we conclude that the samples generated by Borealis are more likely to have come from the ground truth than from the adversarial distribution corresponding to the first four spoofers (1)–(4); the greedy adversary (5) can generate samples mimicking the ground truth but there is no known expression or algorithm to obtain the ‘greedy probability distribution’, thus we cannot use it to generate a Bayesian score. One can see in Fig. 3b that the Bayesian log average is strictly above zero for all remaining adversaries.

**Fig. 4:**
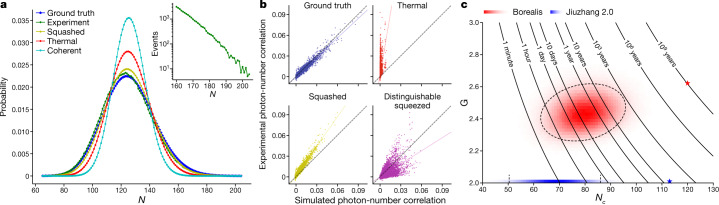
Quantum computational advantage. **a**, Measured photon statistics of 10^6^ samples of a high-dimensional Gaussian state compared with those generated numerically from different hypotheses. The inset shows the same distribution in a log scale having significant support past 160 photons, up to 219. **b**, Scatter plot of two-mode cumulants *C*_*ij*_ for all the pairs of modes comparing experimentally obtained ones versus the ones predicted by four different hypotheses. A perfect hypothesis fit (shown in plot) would correspond to the experimentally obtained cumulants lying on a straight line at 45° (shown in plot). Note that the ground truth is the only one that explains the cumulants well. Moreover, to make a fair comparison all the hypothesis have exactly the same first-order cumulants (mean photon in each mode). **c**, Distribution of classical simulation times for each sample from this experiment, shown as Borealis in red and for Jiuzhang 2.0 in blue^2^. For each sample of both experiments, we calculate the pair (*N*_*c*_, *G*) and then construct a frequency histogram populating this two-dimensional space. Note that because the samples from Jiuzhang 2.0 are all threshold samples they have *G* = 2, whereas samples from Borealis, having collisions and being photon-number resolved, have *G* ≥ 2. Having plotted the density of samples for each experiment in (*N*_*c*_, *G*) space, we indicate with a star the sample with the highest complexity in each experiment. For each experiment, the starred sample is at the very end of the distribution and occurs very rarely; for Jiuzhang 2.0 this falls within the line *G* = 2. Finally, we overlay lines of equal simulation time as given by equation (4) as a function of *N*_*c*_ and *G*. To guide the eye we also show boundaries delineating two standard deviations in plotted distributions (dashed lines).

Finally, we consider the regime of many modes and large photon number, in which calculating the probability of even a single event using a classical computer is unfeasible. In this regime we consider the first- and second-order cumulants of the photon-number distributions of 216 modes and 10^6^ samples against the lossy ground truth and the different spoofer distributions. Note that these samples are generated from the same family of unitaries as the samples generated in the intermediate regime, we only change the brightness of the squeezed input light. In Fig. 4a we plot the total photon-number probability distributions measured in the experiment, and calculated from the ground truth and different spoofers. By construction, the samples generated from each classical adversary have the same first-order cumulants (mode photon-number means) as the ground truth and thus they also have the same total mean photon number centred at $\bar{N}=125$. Deliberately matching the first moments exactly to the ground truth ensures that we give our adversaries fair conditions to spoof our experiment. However, their second-order cumulants, defined between mode *i* and mode *j* as ${C}_{ij}=\langle {n}_{i}{n}_{j}\rangle -\langle {n}_{i}\rangle \langle {n}_{j}\rangle$ with *n*_*i*_ the photon number in mode *i*, are different. We calculate the distribution of all *C*_*ij*_ obtained experimentally and compare the result with those obtained from theoretical predictions and different adversaries, as shown in Fig. 4b. These cumulants can be calculated efficiently. Overall, it is clear that the statistics of experimental samples diverge from the adversarial hypotheses considered and agree with the ground truth of our device (as seen in the top left panel of Fig. 4b) where they cluster around the identity line at 45°.

Unlike earlier experiments^1,2^ in which more than half of the input ports of the interferometer are empty, in this current work every input port of the time-domain interferometer is populated with a squeezed state. This property indicates that the third- and fourth-order photon-number cumulants with no modes repeated are extremely small (≈10^−6^) in our ground truth. The greedy spoofer we implemented using first- and second-order cumulant information automatically produces third-order cumulants on the order of 10^−5^, and thus no extra gain can be attained by using a greedy algorithm with third-order correlations, as they are well explained using only single-mode and pairwise correlations. Note that the difference between the ground truth cumulants and the ones from the greedy samples are more than accounted for by finite size statistics.

For Gaussian states undergoing only common loss (including the special case of lossless GBS), it is straightforward to show that the third-order photon-number cumulants involving any three distinct modes are all strictly zero. Thus, the fact that significant third- and fourth-order cumulants are observed in refs. ^1,2^ is simply a reflection of the fact that most of their inputs are vacuum and that their experiment lacks photon-number resolution. The latter observation could in principle be exploited by a classical adversary to speed up the simulation of GBS with mostly vacuum inputs because strategies exist to speed up the simulation of GBS when the number of input squeezed states is fixed and is a small fraction of the total number of photons observed. These strategies used the fact that hafnians of low-rank matrices^32,33^ can be calculated faster than hafnians of full rank matrices of equal size. For our system, the matrices needed for simulation are all full rank as every input is illuminated with squeezed light.

Finally, note that in Fig. 4b, we do not compare against the cumulants of the greedy sampler. These are, by construction, very close to the ground truth (see details in Supplementary Information). But for the brightnesses for which one calculates cross entropy, they do not perform as well as the samples from our machine.

In the experimental distribution of the total photon number in Fig. 4a, the outcome with the highest probability is *N* = 124.35 ± 0.02 and the distribution has significant support past 160 photons as shown in the inset. The best-known algorithm to simulate GBS^30,34^ scales with the total number of modes and the time it takes to calculate a probability amplitude of a pure-state GBS instance. Thus we can estimate the time it would take to simulate a particular sample *S* = (*n*_1_, …, *n*_*m*_) in Fugaku, the current most powerful supercomputer in the world^35^, to be

4 $${\rm{time}}({N}_{c},G)=\frac{1}{2}{c}_{{\rm{Fugaku}}}M{N}_{c}^{3}{G}^{{N}_{c}/2},$$

where the collision parameter is $G={({\prod }_{i=1}^{M}({n}_{i}+1))}^{1/{N}_{c}}$, *n*_*i*_ is the number of photons in the *i*th mode and *N*_*c*_ is the number of non-zero detector outcomes. We estimate *c*_Fugaku_ = *c*_Niagara_/122.8 from the LINPACK benchmark (a measure of a computer’s floating-point rate of execution) ratio of floating operations per second measured on Fugaku and Niagara^5^ found *c*_Niagara_ = 5.42 × 10^−15^ s from which we get *c*_Fugaku_ = 4.41 × 10^−17^ s. Finally, we take *M* = 216 for both our system and the experiment in ref. ^2^. This assumption slightly overestimates the time it takes a supercomputer to simulate the experiment of ref. ^2^, as it has two-thirds the number of modes of the largest Borealis instance we consider but simplifies the analysis.

Equation (4) captures the collision-free complexity of the hafnian of an *N* × *N* matrix of $O({N}_{c}^{3}{2}^{{N}_{c}/2})$ because in that case *G* = 2. For the purposes of sampling, a threshold detection event that in an experiment can be caused by one or many photons, can always be assumed to have been caused by a single photon, thus threshold samples have the same complexity as in the formula above with *G* = 2 (ref. ^30^), which is quadratically faster than the estimates in refs. ^1,2,36^. One could hope that tensor networks techniques^37^ could speed up the simulation of a circuit such as the one we consider here, but this possibility is ruled out in ref. ^5^ where it is shown that, even when giving tensor network algorithms effectively infinite memory, they require significantly more time than hafnian based methods to calculate probability amplitudes.

On the basis of these assumptions we estimate that, on average, it would take Fugaku 9,000 years to generate one sample, or 9 billion years for the million samples we collected from Borealis. Using the same assumptions, we estimate that Fugaku would require 1.5 h, on average, to generate one sample from the experiment in ref. ^2^, or 8,500 years for the 50 million generated in their experiment. In Fig. 4c, we plot the distribution of classical runtimes of Fukagu for each sample drawn in the experiment, and show the sample with the largest runtime as a star. For comparison, we also compare to the highest brightness experiment from Jiuzhang 2.0 (ref. ^2^). The regime we explore in our experiment is seven orders of magnitude harder to simulate than previous experiments and, moreover, we believe it cannot be spoofed by current state-of-the-art greedy algorithms or classical-Gaussian states in cross entropy.

## Discussion and outlook

We have successfully demonstrated quantum computational advantage in GBS using a photonic time-multiplexed machine. Unlike previous photonic devices used for such demonstrations, Borealis offers dynamic programmability over all gates used, shows true photon-number-resolved detection and requires a much more modest number of optical components and paths. Among all photonic demonstrations of quantum computational advantage–photonic or otherwise–our machine uses the largest number of independent quantum systems: 216 squeezed modes injected into a 216-mode interferometer having three-dimensional connectivity, with up to 219 detected photons. Our demonstration is also more resistant to classical spoofing attacks than all previous photonic demonstrations, enabled by the high photon numbers and photon-number resolution implemented in the experiment.

The programmability and stability of our machine enables its deployment for remote access by users wishing to encode their own gate sequences in the device. Indeed, the machine can be accessed by such users without any knowledge of the underlying hardware, a key property for exploring its use at addressing problems on structured, rather than randomized data. Furthermore, besides demonstrating variable beam-splitting and switching (both in the loops and demultiplexing system), the successful use in our machine of several phase-stabilized fibre loops to act as effective buffer memory for quantum modes is a strong statement on the viability of this technique, which is a requirement in many proposed architectures for fault-tolerant photonic quantum computers^9–11,38^. Our demonstration thus marks a significant advance in photonic technology for quantum computing.

## Methods

### Optical circuit

The input of the interferometer is provided by a single optical parametric oscillator (OPO), emitting pulsed single-mode squeezed states at a 6 MHz rate that are then sent to three concatenated, programmable, loop-based interferometers. Each loop contains a VBS, including a programmable phase shifter, and an optical fibre delay line acting as a buffer memory for light, and allows for the interference of modes that are temporally adjacent (*τ* = (6 MHz)^−1^), or separated by six or 36 time bins (6 *τ* or 36 *τ*) in the first, second and third loop, respectively. Optical delays provide a compact and elegant method to mediate short- and long-range couplings between modes. The high-dimensional Gaussian state generated for this experiment can be visualized, as depicted above the three loops in Fig. 1, using a three-dimensional lattice representation. Given a lattice of size *a* = 6, where *a* is the number of modes separating two interacting pulses in the second loop, one can form a cubic lattice by injecting *M* = *a*^3^ = 216 squeezed-light pulses into the interferometer.

Owing to the use of a single time-multiplexed squeezed-light source, all temporal modes are, to very good approximation, indistinguishable in all degrees of freedom except time signature, and passively phased locked with respect to each other; the squeezer is driven by pump pulses engineered to generate nearly single-temporal-mode squeezed-light pulses on a 6 MHz clock. Spatial overlap is ensured by using single-mode fibre coupling at the entrance and exit of each loop delay, and samples are collected using an array of photon-number resolving (PNR) detectors based on superconducting transition-edge sensors (TES) with 95% detection efficiency^39,40^. These samples consist of 216 (integer) photon-number measurement outcomes for as many modes. To bridge the gap between the 6 MHz clock, chosen to maintain manageable fibre loop lengths, and the slower relaxation time of the TES detectors, a 1-to-16 binary-tree switching network was used to partially demultiplex the pulse train after the loops and before the detectors.

### Experimental challenges

Despite the simple conceptual design of Borealis (Fig. 1), building a machine capable of delivering quantum computational advantage in a programmable fashion using photonics, in a large photon-number regime, required solving considerable technological hurdles that were previously outstanding. These include: (1) lack of PNR-compatible single-mode squeezed-light sources and non-invasive phase stabilization techniques requiring bright laser beams, (2) slow PNR reset times that would necessitate unfeasibly long fibre loops and (3) lack of sufficiently fast and low-loss electro-optic modulators (EOMs) preventing programmability. Our solutions to these challenges for this work are, respectively, (1) the design of a bright and tunable source of single-mode squeezed states and phase stabilization techniques (OPO and interferometer) using locking schemes compatible with PNR detectors, (2) active demultiplexing to increase the effective rate of PNR acquisition by a factor of 60, compared to previous systems^40^, by constructing a low-loss 1-to-16 binary switch tree and developing new photon-number extraction techniques and (3) the use of new, efficient and fast customized EOMs (QUBIG GmbH) that enable arbitrary dynamic programming of photonic gates with low loss and high speeds. The success of this experiment also relies on a robust calibration routine, accurately extracting all experimental parameters contained in the transfer matrix *T* and the squeezing parameters *r* that define each GBS instance. We describe each of these advances in the following sections. Other details pertinent to the apparatus can be found in the Supplementary Information.

With further fabrication and device optimization, the raw operational speed of PNR detectors can be increased, eliminating the need for the demultiplexer (demux) and associated losses (roughly 15%). Improvements to the filter stack (20% loss) would also considerably increase performance. Several paths thus exist to even further increase the robustness of our machine against hypothetical improved classical adversaries. In addition, in trial runs we have extended the number of accessible modes to 288 (see Supplementary Information) without any changes to the physical architecture, and expect further scalability in this number to be readily achievable by improving the long-time stabilization of the device. Such scaling will place the device even further ahead of the regime of classical simulability and potential vulnerability to spoofing.

For applications requiring a universal interferometer, a recirculation loop long enough to accommodate all 216 modes could be implemented^41^, replacing any two of the three existing loops. The remaining existing loop would be nested in the larger 216-mode loop, allowing repeated application of the remaining VBS to all 216 modes, albeit at the cost of higher losses.

### Pulsed squeezed-light source

The main laser is an ultralow phase noise fibre laser with a sub-100 Hz linewidth centred at 1,550 nm, branched out into different paths. To prepare the pump, in one path pulses are carved using a 4 GHz lithium niobate electro-optic intensity modulator. It is then amplified and upconverted to 775 nm using a fibre-coupled MgO:LN ridge waveguide. The resulting pump is a 6 MHz stream of 3-ns-duration rectangular pulses with an average power of 3.7 mW. Squeezed-light pulses are generated in a doubly resonant, phase-stabilized hemilithic cavity^42^ comprising a 10-mm-long plano-convex potassium titanyl phosphate crystal with its temperature stabilized at 32.90 °C using a Peltier element, for optimal Type-0 phase matching (Supplementary Information). All spectral side bands of the OPO cavity, around the degenerate frequency band, are suppressed by more than 25 dB using a pair of fibre Bragg gratings (0.04 nm bandwidth at 0.5 dB), one in reflection and the other in transmission (more details in Supplementary Information).

### Programmable photonic processor

A train of single-mode squeezed vacuum pulses is emitted by the OPO, coupled into a single-mode fibre and directed towards the programmable photonic processor consisting of three loop-based interferometers in series, as shown in Fig. 1. Each loop ${\ell }=0,1,2$ is characterized by a VBS with transfer matrix

5 $$B{S}^{{\ell }}({\alpha }_{k},{\varphi }_{k})=(\begin{array}{cc}{e}^{i{\varphi }_{k}}\,\cos \,{\alpha }_{k} & i\sqrt{{\eta }_{{\ell }}}{e}^{i{\mu }_{{\ell }}}\,\sin \,{\alpha }_{k}\\ i{e}^{i{\varphi }_{k}}\,\sin \,{\alpha }_{k} & \sqrt{{\eta }_{{\ell }}}{e}^{i{\mu }_{{\ell }}}\,\cos \,{\alpha }_{k}\end{array})$$

where each phase *ϕ*_*k*_ = [−*π*/2, *π*/2] and *α*_*k*_ = [0, *π*/2] can be programmed independently, ${\mu }_{{\ell }}$ is a phase offset associated with each loop and ${\eta }_{{\ell }}$ is the energy transmittance coefficient associated with one complete circulation in loop ${\ell }$. The time delay experienced in the first loop is *τ* = 1/(6 MHz), equals the delay between two consecutive squeezed-light pulses, whereas the second and third loops have 6 *τ* and 36 *τ* time delay, respectively. The transmittance *t*_*k*_ of a VBS with parameter *α*_*k*_ is given by *t*_*k*_ = cos^2^*α*_*k*_. For *t*_*k*_ = 1 all the incoming light is directed into the fibre delay, whereas the light entering the VBS from the fibre delay is fully coupled out. The output of the last loop is coupled into a single-mode fibre and directed towards the final sampling stage of the experiment.

All three loops are independently phase stabilized using a counter-propagating laser beam, piezo transducers and lock-in techniques. To avoid stray light from reflections of this beam towards the detectors, we alternate between measurement (65 μs) and phase stabilization of the loops (35 μs), leading to a sampling rate of 10 kHz. The estimated phase noise (standard deviation from the mean) inside the interferometer is 0.02, 0.03 and 0.15 rad for the first, second and third loops, respectively, as measured with classical pulses. We carefully reduced mode mismatch throughout the entire interferometer: spatial overlap is ensured using single-mode fibres, with coupling efficiencies >97%, and the length of each loop delay is carefully adjusted to have >80% classical visibility between 250-ps-long classical pulses, which gives >99% temporal overlap for the squeezed states.

### Connectivity

The programmable time-domain multiplexed architecture implemented here and introduced in ref. ^5^ generates sufficiently connected transmission matrices (in which two-thirds of the entries of the matrix are non-zero) to furnish a high level of entanglement between the modes (we estimate the log negativity between modes 0…*i*−1 and *i*…216 for the ground truth to be on average 5.96 for $i\in \{36,72,108,144,180\}$), while keeping losses sufficiently low (with transmission above 33%). This is not the case for other architectures in which one either has to give up programmability^1,2^ or suffer steep losses that, in the asymptotic limit of many modes, render the sampling task roughly simulable as the loss scales exponentially with the system size^31^. In a universal programmable interferometer each mode passes through several lossy components (with transmission *η*_unit_) proportional to the number of modes. For the interferometers considered here, each mode sees a fixed number (six) of beamsplitters in which the loss is dominated by the transmission of the largest loop. If the shortest loop, which accommodates only one mode, has transmission *η*_unit_ then the largest loss is given by ${\eta }_{{\rm{unit}}}^{36}$, which should be contrasted with ${\eta }_{{\rm{unit}}}^{216}$ for a universal interferometer. Whereas we sacrifice some connectivity, the many-mode entanglement predicted in our ground truth (logarithmic negativity^43^ of 6.08 when splitting the modes of the ground truth between the first and last 108) is comparable to the one found in Gaussian state prepared using a random Haar-interferometer with a comparable net transmission and brightness (for which the logarithmic negativity across the same bipartition is 15.22). For the largest experiment considered below, the net transmittance is around 33%. As discussed in the Methods, combined with the high brightness of our source averaging *r* ~ 1.1, places our experiment well beyond any attempt at a now-known polynomial-time approximate classical simulation^31^.

### Sampling of high-dimensional GBS instances

All temporal modes of our synthesized high-dimensional Gaussian states are sampled using superconducting TES allowing photon-number resolution up to 13 photons per detector in our data. Relaxation time of our TES, back to baseline following illumination, is of the order of 10 to 20 μs corresponding to 50–100 kHz (ref. ^40^), and depends on the expected photon number. At this speed, the length of the shortest loop delay would be 2 km, leading to excessive losses and more challenging phase stabilization in our system. Thus our pulse train and thus processing speed of 6 MHz, chosen to maintain manageable loop lengths, is too fast for a reliable photon-number extraction. To bridge the gap between the typical PNR speed and our processing speed, we use a demultiplexing device allowing to speed up by effectively 16×, and to develop a postprocessing scheme, described below, for ‘tail-subtraction’ enabling operation of each PNR at 375 kHz.

The role of the demux, depicted as a binary tree in Fig. 1, is to reroute squeezed-light pulse modes from the incoming train into 16 separate and independent spatial modes, each containing a fibre-coupled PNR-TES detector. There are 15 low-loss resonant EOMs grouped in four different layers. EOMs in each layer have a preset frequency: one at 3 MHz, two at 1.5 MHz, four at 750 kHz and eight at 375 kHz. Each EOM is sandwiched between two polarizing beamsplitter and a quarter-waveplate at 45° in front. The modulators are driven by a standalone unit, generating several phase-locked sine wave signals temporally synchronized with the input train. The switching extinction ratio is measured to be above 200:1 for all modulators.

Several methods have been demonstrated to extract photon numbers from a PNR’s output voltage waveform, each with their own advantages^44–47^. Here we use a modified version of the method presented in ref. ^47^. First, each detector is calibrated using well separated pulses of squeezed light with a high mean photon number around $\langle n\rangle \approx 1$ and 500 × 10^3^ repetitions. This gives enough high photon-number events to ensure that at least the 0 to 11 photon clusters can be identified using the area method. From each cluster, the mean shape of the waveforms is defined. To extract the photon-number arrays from experiment, the mean square distance between each waveform and the mean shape is estimated. The photon number is then assigned to the closest cluster. Because we operate the individual PNRs at 375 kHz, faster than the relaxation time (back to baseline following illumination), the tail of each pulse still persists when the next pulse arrives at the same PNR. To avoid these tails reducing photon-number extraction fidelity in a pulse, the mean shape for the identified previous photon number is subtracted. See Supplementary Information for details.

### Estimation of the ground truth parameters

Given that all the squeezed states come from the same squeezer and the programmability of our system, we can parametrize and characterize the loss budget of our system using a very small set of parameters. The first set of parameters correspond to the relative efficiencies of the 16 different demux-detector channels, *η*_demux,*i*_ for $i\in \{0,1,\ldots ,15\}$. The second parameter is simply the common transmittance *η*_*C*_. Finally, we have the transmittance associated with a round-trip through each loop *η*_*k*_ for $k\in \{0,1,2\}$.

To characterize the first two parameter sets, namely the demux and common loss, we set all the loops to a ‘bar’ state (*α*_*k*_ = *π*/2), preventing any light from entering the delays. As the input energy is the same, we can simply estimate the ratio of the transmittance of the different demux-detector channels as ${\eta }_{{\rm{demux}},i}/{\eta }_{{\rm{demux}},j}={\bar{n}}_{i}/{\bar{n}}_{j}$ where ${\bar{n}}_{j}$ is the mean photon number measured in the detector *j*. Without loss of generality, we can take the largest of the *η*_demux,*i*_ to be equal to one and assign any absolute loss from this and any other channel into the common loss *η*_*C*_. To determine the common loss, we use the noise reduction factor (NRF), defined as^48,49^

6 $${\rm{NRF}}=\frac{{\Delta }^{2}({n}_{i}-{n}_{j})}{\langle {n}_{i}+{n}_{j}\rangle },$$

where *n*_*i*_ and *n*_*j*_ are the photon-number random variables measured in mode *i* and *j*, and we write variances as ${\Delta }^{2}X={\langle X\rangle }^{2}-{\langle X\rangle }^{2}$.

If losses can be considered as uniform, which is an excellent approximation if we use only the loop with the shortest delay, it is straightforward to show that the NRF of a two-mode squeezed vacuum gives directly the loss seen by the two modes as NRF_TMSV_ = 1−*η*. To prepare the two-mode squeezed vacuum we set our VBS matrix to be proportional to $$(\begin{array}{ll}1 & i\\ i & 1\end{array})$$ when the two single-mode squeezed pulses meet at the beamsplitter. To this end, we use the following sequence [*t*_0_ = 0, *t*_1_ = 1/2, *t*_2_ = 0], where, recall, we write *t*_*i*_ = cos^2^*α*_*i*_ to indicate the transmittance of a particular loop time bin *i*. We can now scan the controllable phase of the VBS, *ϕ*_*k*_, and determine where the minimum occurs $({\varphi }_{k}^{{\rm{\min }}}={\mu }_{0}\,{\rm{mod}}\,\pi )$, and at the same time provide the relative offset in the first loop and the net transmittance of the setup. This observation can be used to obtain the phase offset of any other loop round-trip. Although in the current version of our system these are set by the locking system, they can in principle also be made programmable. The transmittance *η* = 1 − NRF_TMSV_ = *η*_*C*_ × *η*_0_ × *η*_demux_ is the product of the common transmittance *η*_*C*_, the round-trip in the first loop *η*_0_ and the average transmittance associated with two demux-detector channels used to detect the two halves of the two-mode squeezed vacuum ${\eta }_{{\rm{demux}}}=\frac{1}{2}\{{\eta }_{{\rm{demux}},i}+{\eta }_{{\rm{demux}},j}\}$. From this relation, we can find

7 $${\eta }_{C}=\frac{1-{{\rm{NRF}}}_{{\rm{TMSV}}}}{{\eta }_{0}\times {\eta }_{{\rm{demux}}}}.$$

This calibration depends on knowing the value of the round-trip transmittance factor associated with the first loop. To estimate the round-trip transmittance of a particular loop ${\ell }$, we bypass the other loop delays and compare the amount of light detected when light undergoes a round-trip through a particular loop, relative to when all the round-trip channels are closed, that is, all loops in a ‘bar’ state. We obtain ${\eta }_{{\ell }}$, which we can then plug in equation (7) to complete the calibration sequence.

Finally, having characterized the loss budget in the experiment, we can obtain the brightness and squeezing parameters at the source by measuring photon numbers when all the loops are closed and then dividing by the net transmittance. For any of the three regimes considered in the main text the standard deviation of the estimated squeezing parameters and mean photon numbers is below 1% of the respective means.

From the same data acquired above for a pair of modes, we calculate the unheralded second-order correlation

8 $${g}^{(2)}=\frac{\langle {n}_{i}^{2}\rangle -\langle {n}_{i}\rangle }{{\langle {n}_{i}\rangle }^{2}}$$

for each pair of temporal modes. When we attain the minimum NRF at *ϕ*_*k*_ = *μ*_0_, that is, when we prepare two-mode squeezed vacuum, it is easy to see that^50^

9 $${g}^{(2)}=1+\frac{1}{K},$$

where *K* is the so-called Schmidt number of the source. This quantifies the amount of spectral mixedness in the generated squeezed light. An ideal squeezed vacuum light source would yield *g*^(2)^ = 2. We report *K* = 1.12 for *g*^(2)^ = 1.89 for the dataset used in the large mode and photon-number regime.

### Theory sections

#### Transfer matrix, *T*

The loop-based interferometer, as well as any other interferometer, can be described by a transfer matrix *T* that uniquely specifies the transformation effected on the input light. For our GBS implementation, this interferometer is obtained by combining three layers of phase gates and beamsplitters (two-mode gates), interfering modes that are contiguous, or separated by six or 36 time bins, which we write as

10 $$T=\sqrt{{\eta }_{C}}{T}_{{\rm{demux}}}[\mathop{\mathop{\otimes }\limits_{d=0}}\limits^{D-1}\mathop{\mathop{\otimes }\limits_{i=0}}\limits^{M-{a}^{d}}{B}_{i,i+{a}^{d}}({{\rm{VBS}}}^{d}({\alpha }_{i},{\varphi }_{i}))]$$

where in our case *D* = 3 gives the number of loops, while ${a}^{d}{|}_{d\in \{0,1,2\}}=$ {1, 6, 36} with *a* = 6 gives the number of modes that each loop can hold. ${B}_{i,i+{a}^{d}}$(VBS) is an *M* × *M* transfer matrix that acts like the VBS in the subspace of modes *i* and *j* = *i* + *a*^*d*^ and like the identity elsewhere.

In the last equation, *η*_C_ is the common transmittance throughout the interferometer associated with the escape efficiency of the squeezer cavity and the propagation loss in common elements. *T*_demux_ is a diagonal matrix that contains the square roots of the energy transmittance into which any of the modes are rerouted for measurement using the demux. Because the demux has 16 channels, it holds that ${({T}_{{\rm{demux}}})}_{i,i}={({T}_{{\rm{demux}}})}_{i+16,i+16}=\sqrt{{\eta }_{{\rm{demux}},i}}$. Finally, we set the phases of the VBS to be uniformly distributed in the range [−*π*/2, *π*/2] and the transmittances to be uniformly in the range [0.45, 0.55]. This range highlights the programmability of the device while also generating high degrees of entanglement that are typically achieved when the transmittance is half.

In the idealized limit of a lossless interferometer, the matrix representing it is unitary, otherwise the matrix *T* is subunitary (meaning its singular values are bounded by 1). The matrix *T* together with the input squeezing parameters *r* defines a GBS instance. Squeezed states interfered in an interferometer (lossy or lossless) always lead to a Gaussian state, that is, one that has a Gaussian Wigner function. Moreover, loss is never able to map a non-classical state (having noise in a quadrature below the vacuum level) to a classical state. Thus there exists a finite separation in Hilbert space between lossy-squeezed states and classical states. To gauge this separation, and how it influences sampling, we use the results from ref. ^31^ to show in the section ‘Regimes of classical simulability’ that the probability distribution associated with the ground truth programmed into the device cannot be well-approximated by any classical-Gaussian state.

Similar to previous GBS experiments in which the ground truth to which a quantum computer is compared contains imperfections due to loss, we also benchmark our machine against the operation of a lossy unitary. In this more realistic scenario in which losses are included, the state generated at the output cannot be described by a state vector and thus one cannot assign probability amplitudes to an event. In this case, probabilities are calculated from the density matrix of the Gaussian state using the standard Born rule and then the probability of an *N* photon event is proportional to the hafnian of a 2*N* × 2*N* matrix.

#### Regimes of classical simulability

As a necessary but not sufficient test for beyond-classical capabilities of our machine, we consider the GBS test introduced in ref. ^31^. This test states that a noisy GBS device can be classically efficiently simulated up to error *ϵ* if the following condition is satisfied:

11 $$\text{sec}{\rm{h}}\left\{\frac{1}{2}\,\max \left[0,\,\mathrm{ln}\,\frac{1-2{q}_{D}}{\eta {e}^{-2r}+1-\eta }\right]\right\} > {e}^{-\frac{{{\epsilon }}^{2}}{4M}}.$$

Here *q*_D_ is the dark count probability of the detectors, *η* is the overall transmittance of the interferometer, *r* is the squeezing parameter of the *M* input squeezed states (assumed to be identical) and *ϵ* is a bound in the TVD of the photon-number probability distributions of GBS instance and the classical adversary. For our experiment, we estimate an average transmittance of *η* = Tr(*TT*^†^)/M = 0.32, *q*_D_ = 10^−3^, an average squeezing parameter of *r* = 1.10 and *M* is the total number of modes. With these parameters we find that the inequality above has no solution for ${\epsilon }\in [0,1]$, meaning that our machine passes this non-classicality test.

#### Greedy adversarial spoofer

The greedy adversarial spoofer tries to mimic the low order correlations of the distribution and takes as input the *k* order, $k\in \{1,2\}$, marginal distributions and optimizes a set of samples (represented as an array of size *M* × *K*) so as to minimize the distance between the marginals associated with this array and the ones associated with the ground truth. In a recent preprint Villalonga et al.^3^ argue that, using a greedy algorithm such as the one just described, they can obtain a better score at the cross-entropy benchmark against the ground truth of the experiment in refs. ^1,2^ than the samples generated in the same experiment. We generalized the greedy algorithm introduced by Villalonga et al.^3^ to work with photon-number-resolved samples and find that it is unable to spoof the samples generated by our machine at the cross-entropy benchmark that we use for scoring the different adversaries. Details of the algorithm are provided in the Supplementary Information.

## Online content

Any methods, additional references, Nature Research reporting summaries, source data, extended data, supplementary information, acknowledgements, peer review information; details of author contributions and competing interests; and statements of data and code availability are available at 10.1038/s41586-022-04725-x.

## Supplementary information

Supplementary Information.

## Data Availability

The datasets generated and analysed for this study are available from this link: https://github.com/XanaduAI/xanadu-qca-data.
