# Supplementary material for: Quantum computational advantage with a programmable photonic processor
Source: Nature. 2022 Jun 1;606(7912):75–81. doi: 10.1038/s41586-022-04725-x (PMC9159949; doi:10.1038/s41586-022-04725-x)
Supplement: Supplementary file 1 — Supplementary Information. [file 41586_2022_4725_MOESM1_ESM.pdf]

nature portfolio

https://doi.org/10.1038/s41586-022-04725-x

Supplementary information

Quantum computational advantage with a programmable photonic processor

In the format provided by the authors and unedited

Quantum computational advantage with a programmable photonic processor
Supplementary Information

L.S. Madsen,* F. Laudenbach,* M.F. Askarani,* F. Rortais, T. Vincent, J.F.F. Bulmer, F.M. Miatto, L. Neuhaus, L.G. Helt, M.J. Collins, V.D. Vaidya, M. Menotti, I. Dhand, Z. Vernon, N. Quesada,† and J. Lavoie‡
Xanadu, Toronto, ON, M5G 2C8, Canada

A.E. Lita, T. Gerrits, and S.W. Nam
National Institute of Standards and Technology, Boulder, CO, USA

CONTENTS

1. Gaussian Boson Sampling and adversaries 3
 1.1. Generating samples for the classical adversaries 6
  1. 3rd order greedy 9
 1.2. TVD for classical adversaries in a 16-mode GBS instance 11
 1.3. Numerical specifications of the classical adversaries 13

2. Experimental setup and methods 15
 2.1. Squeezed-light source 15
 2.2. Loop-based programmable interferometer 16
  1. Implementation errors of gate parameters 19
  2. Phase stabilization of the interferometers 21
 2.3. 1-to-16 demultiplexer 23
 2.4. Photon number resolving detection 23
 2.5. System transmittance 26
 2.6. Generation and evaluation of two-mode squeezed states 26

3. Unitary matrix 28

4. Benchmarking methods 30
 4.1. Obtaining the transfer matrix 30
 4.2. Photon-number statistics 32

5. Scalability 33

Note on commercial equipment 34

References 35

* These authors contributed equally.
† nicolas.quesada@polymtl.ca
‡ jonathan@xanadu.ai

1. GAUSSIAN BOSON SAMPLING AND ADVERSARIES

In Gaussian Boson Sampling a set of single-mode gaussian states – quantum states having a Gaussian Wigner function – are sent into an interferometer and then measured using photon-counting detectors. The input Gaussian states are each specified by a 2×2 covariance matrix that can be assumed to be diagonal without loss of generality, and a length-two vector of means. In the main text we consider the following families of states specified by a quadrature covariance matrix and a vector of means:

$$\boldsymbol{\sigma}_{\text{squeezed}} = \frac{\hbar}{2}\begin{pmatrix} e^{-2r} & 0 \\ 0 & e^{2r} \end{pmatrix}, \quad \boldsymbol{\mu}_{\text{squeezed}} = (0,0), \tag{1}$$

$$\boldsymbol{\sigma}_{\text{thermal}} = \frac{\hbar}{2}\begin{pmatrix} 2\bar{n}+1 & 0 \\ 0 & 2\bar{n}+1 \end{pmatrix}, \quad \boldsymbol{\mu}_{\text{thermal}} = (0,0), \tag{2}$$

$$\boldsymbol{\sigma}_{\text{squashed}} = \frac{\hbar}{2}\begin{pmatrix} 1 & 0 \\ 0 & 4\bar{n}+1 \end{pmatrix}, \quad \boldsymbol{\mu}_{\text{squashed}} = (0,0), \tag{3}$$

$$\boldsymbol{\sigma}_{\text{coherent}} = \frac{\hbar}{2}\begin{pmatrix} 1 & 0 \\ 0 & 1 \end{pmatrix}, \quad \boldsymbol{\mu}_{\text{coherent}} = \sqrt{\frac{\hbar}{2}}(\Re(\beta), \Im(\beta)). \tag{4}$$

Thermal and squashed states are parametrized in terms of their mean photon number $\bar{n}$, while the mean photon numbers of the squeezed and coherent states are given by $\sinh^2 r$ and $|\beta|^2$ respectively in terms of their squeezing parameter r and complex-displacement β. Given the covariance matrices and vectors of means of a set of modes input into a interferometer we can construct the output covariance matrix and vector of means of the collection of modes. Note that the only non-classical states from these families are the squeezed states that have one quadrature variance below the vacuum level $\frac{\hbar}{2}$. The moments of the different input states can also be specified using the moments of the annhilation and creation operators. The first order moments are simply $\alpha \equiv \langle a \rangle$ and the second order ones are $n = \langle a^\dagger a \rangle - |\alpha|^2$ and $m = \langle a^2 \rangle - \alpha^2$.

For the families of Gaussian states above we have

$$n_{\text{squeezed}} = \sinh^2 r, \quad m_{\text{squeezed}} = \tfrac{1}{2}\sinh 2r, \quad \alpha_{\text{squeezed}} = 0, \tag{5}$$

$$n_{\text{thermal}} = \bar{n}, \quad m_{\text{thermal}} = 0, \quad \alpha_{\text{thermal}} = 0, \tag{6}$$

$$n_{\text{squashed}} = \bar{n}, \quad m_{\text{squashed}} = \bar{n}, \quad \alpha_{\text{squashed}} = 0, \tag{7}$$

$$n_{\text{coherent}} = 0, \quad m_{\text{coherent}} = 0, \quad \alpha_{\text{coherent}} = \beta. \tag{8}$$

In the Heisenberg picture, the action of the interferometer $\boldsymbol{T}$ transforms the input annihilation operator of the modes a_j according to

$$a_i \to a_i' = \sum_{j=1}^{M} T_{ij} a_j + \sum_{j=1}^{M} \left(\sqrt{\mathbb{I}_M - \boldsymbol{T}\boldsymbol{T}^\dagger}\right)_{ij} e_j. \tag{9}$$

These operators satisfy the canonical commutation relations $[a_i, a_j] = [a_i^\dagger, a_j^\dagger] = 0$ and $[a_i, a_j^\dagger] = \delta_{ij}$. Similarly, the operators e_j represent bosonic environmental modes into which photons from the input modes can be lost. Note that if the transfer matrix $\boldsymbol{T}$ is unitary ($\boldsymbol{T}\boldsymbol{T}^\dagger = \mathbf{I}_M$) then the modes of interest decouple from the environment modes giving a lossless interferometer.

For a set of input states entering an interferometer we can easily write how their first and second quadrature moments transform. If we denote

$$\alpha_i = \langle a_i \rangle, \tag{10}$$

$$n_{ij} = \langle a_i^\dagger a_j \rangle - \alpha_i^* \alpha_j, \tag{11}$$

$$m_{ij} = \langle a_i a_j \rangle - \alpha_i \alpha_j, \tag{12}$$

the input moments $\boldsymbol{\alpha}^{\text{in}}, \boldsymbol{n}^{\text{in}}$ and $\boldsymbol{m}^{\text{in}}$ are transformed according to

$$\boldsymbol{\alpha}^{\text{in}} \to \boldsymbol{\alpha}^{\text{out}} = \boldsymbol{T}\boldsymbol{\alpha}^{\text{in}}, \tag{13}$$

$$\boldsymbol{n}^{\text{in}} \to \boldsymbol{n}^{\text{out}} = \boldsymbol{T}^* \boldsymbol{n}^{\text{in}} \boldsymbol{T}^T, \tag{14}$$

$$\boldsymbol{m}^{\text{in}} \to \boldsymbol{m}^{\text{out}} = \boldsymbol{T} \boldsymbol{m}^{\text{in}} \boldsymbol{T}^T. \tag{15}$$

In particular if the input is a product state, then the input matrices are diagonal . For a state with zero quadrature mean $\boldsymbol{\alpha} = \boldsymbol{0}$, it is straightforward to write the first two moments of the

photon number distribution

$$\bar{n}_i = \boldsymbol{n}_{ii}, \tag{16}$$

$$\mathrm{Cov}(n_i, n_j) = |\boldsymbol{n}_{ij}|^2 + |\boldsymbol{m}_{ij}|^2 + \delta_{ij}\boldsymbol{n}_{ii}. \tag{17}$$

Higher order moments can be obtained using Wick's theorem [1].

Finally, given a zero-mean ℓ-mode Gaussian state with moment matrices $\boldsymbol{m}$ and $\boldsymbol{n}$, we can write the probability of a photon number measurement with outcome $\boldsymbol{S} = (S_1, S_2, \ldots, S_\ell) \in \mathbb{N}_0^\ell$ in terms of the hafnian function defined as [2, 3]

$$\mathrm{Haf}(A) = \frac{1}{k!2^k} \sum_{\sigma \in \mathrm{S}_{2k}} \prod_{i=1}^{k} A_{\sigma(2i-1),\sigma(2i)}, \tag{18}$$

where S_{2k} is the symmetric group of permutations of $2k$ objects. To write the probability, we define

$$\boldsymbol{Q} = \begin{pmatrix} \boldsymbol{n} & \boldsymbol{m} \\ \boldsymbol{m}^* & \boldsymbol{n}^* \end{pmatrix} + \frac{\mathbb{I}_{2\ell}}{2}, \tag{19}$$

$$\boldsymbol{A} = \boldsymbol{X}_{2\ell}(\mathbb{I}_{2\ell} - \boldsymbol{Q}^{-1}), \tag{20}$$

$$\boldsymbol{X}_{2\ell} = \begin{pmatrix} \boldsymbol{0} & \mathbb{I}_\ell \\ \mathbb{I}_\ell & \boldsymbol{0} \end{pmatrix}, \tag{21}$$

and finally write [4]

$$\Pr(\boldsymbol{S}) = \frac{1}{\sqrt{\det(\boldsymbol{Q})}} \frac{\mathrm{Haf}(\boldsymbol{A}_{\boldsymbol{S}\oplus\boldsymbol{S}})}{\prod_{i=1}^{\ell} S_i!}. \tag{22}$$

In the last equation $\boldsymbol{S} \oplus \boldsymbol{S} = (S_1, \ldots, S_\ell, S_1, \ldots, S_\ell)$ and the matrix $\boldsymbol{A}_{\boldsymbol{K}}$ is constructed from $\boldsymbol{A}$ by repeating the ith row and column of $\boldsymbol{A}$ k_i-many times (e.g., if $k_i = 0$, both the corresponding row and column are removed entirely). Note that if the zero-mean Gaussian state is pure, then we can write $\boldsymbol{n} = \boldsymbol{U}^* \left[\oplus_{i=1}^{\ell} \sinh^2 r_i\right] \boldsymbol{U}^T$ and $\boldsymbol{m} = \boldsymbol{U}\left[\frac{1}{2} \oplus_{i=1}^{\ell} \sinh 2r_i\right] \boldsymbol{U}^T$ with $\boldsymbol{U}$ a unitary matrix and then $\boldsymbol{A} = \boldsymbol{B} \oplus \boldsymbol{B}^*$ with $\boldsymbol{B} = \boldsymbol{U}\left[\oplus_{i=1}^{\ell} \tanh r_i\right] \boldsymbol{U}^T$ and we can write the probability as the

modulus squared of a probability amplitude

$$\Pr(\boldsymbol{S}) = \frac{1}{\sqrt{\det(\boldsymbol{Q})}} \frac{|\mathrm{Haf}(\boldsymbol{B}_{\boldsymbol{S}})|^2}{\prod_{i=1}^{\ell} S_i!}. \tag{23}$$

The complexity of calculating the probability of a pure state photon-number event with outcome $\boldsymbol{S} = (S_1,\ldots,S_\ell)$ is given by $O([\sum_{i=1}^{\ell} S_i]\sqrt{\prod_{i=1}^{\ell}(1+S_i)}$. For mixed states this complexity becomes $O([\sum_{i=1}^{\ell} S_i]\prod_{i=1}^{\ell}(1+S_i)$ using state of the art methods [5]. For threshold GBS with a binary pattern $\boldsymbol{S}$ the complexity is given by the torontonian of a matrix of size given by the number of clicks C. Recent improvements have taken the complexity of this calculation from $C^3 2^C$ as in Ref. [6] to $C^\omega 2^C$ with $\omega \sim 1.1$ as shown in Ref. [7].

1.1. Generating samples for the classical adversaries

Scoring the cross entropy of our experimental samples against the various spoofers requires us to fabricate synthetic samples for each of the hypotheses. The Gaussian hypotheses of squashed, thermal, and coherent states are classical input states and can therefore be sampled from efficiently (in our case using Xanadu's open-source package TheWalrus [8]). The samples generated by distinguishable squeezed states are drawn from a distribution of single-mode squeezed states that have gone through the interferometer individually (experiencing loss and phase but no interference). In the generation of all sets of synthetic samples (including the greedy algorithm samples described in the next section), the mean photon number of each output mode was exactly matched to the ground truth in order to give our adversaries a fair chance to spoof our experimental samples.

We also generalize the greedy algorithm introduced by Villalonga et al. [9] to work with photon-number resolved samples. We note that unlike previous GBS experiments where only a fraction of the input modes of the interferometer are used and only click cumulants for threshold detection events are calculated, our experiment involves counting photons in a setup where every input of the interferometer is illuminated. These differences cause the third and higher order cumulants to be extremely small. In fact, if the input squeezing parameters are all equal and the losses are uniform, one can show that the third-order cumulants, involving three distinct modes, of the ground truth distributions are exactly zero. Finally, we note that Villalonga et al. also use a mean-field solution to a spin-1/2 problem to generate samples using a Boltz-

mann machine. This approach does not generalize in a straightforward manner to sampling problems with more than two outcomes per mode and thus have not been considered here.

We produce a set of D synthetic M-mode samples via a greedy algorithm. The samples are arranged as a $D \times M$ matrix S, where each row S_d is an array of M photon number measurements S_{dm} with values ranging between 0 and some maximum cutoff value (which we set to 10 for the samples considered in the main text). Each integer S_{dm} represents the outcome of a PNR measurement on mode m of an M-mode Gaussian quantum state. The algorithm works by modifying the entries in S so as to decrease the distance from the target 1-body histogram $T^{(1)}_{mj} = \text{int}(DP^{(1)}_{mj})$ (i.e. the expected number of times j photons are measured in mode m) and from the target 2-body histogram $T^{(2)}_{m_1 m_2 j_1 j_2} = \text{int}(DP^{(2)}_{mnjk})$ (i.e. the expected number of times (j_1, j_2) photons are measured in modes (m_1, m_2)). The probability distributions $P^{(1)}$ and $P^{(2)}$ are computed from the Gaussian state directly.

The algorithm works as follows:

1. For each column index $m \in [1, M]$: fill the column with D independent samples from the 1-body target marginal in that mode: $S_{dm} \sim P^{(1)}_m$. At this stage the samples have the correct 1-body marginals, but each mode is sampled independently, and therefore the 2-body marginals are not respected. One could also use the 2-body marginal distribution and generate the samples at modes m_1 and m_1 in pairs: $(S_{dm_1}, S_{dm_2}) \sim P^{(2)}_{m_1 m_2}$. This helps a bit but not much, as only $O(M)$ out of $O(M^2)$ 2-body marginals would be respected.

2. The empirical sample histograms are stored as two arrays: $C^{(1)}_{mj} = \sum_d 1\{S_{dm} = j\}$ and $C^{(2)}_{m_1 m_2 j_1 j_2} = \sum_d 1\{S_{dm_1} = j_1, S_{dm_2} = j_2\}$. The L_1 count distance can now be computed as $\Delta = ||C^{(1)} - T^{(1)}||_1 + ||C^{(2)} - T^{(2)}||_1$ and a TVD proxy can be computed as $\delta = \Delta / D$. We include the 1-body L_1 count distance in the 2nd order sampler in order to help it respect the 1-body marginals even when there are too few samples to determine it accurately from the 2-body counts.

3. Until a stopping criterion is satisfied, loop over columns and rows and replace the value S_{dm} with the value S^*_{dm} that minimizes the L_1 count distance (this is where the algorithm is greedy).

In the algorithm we write $C^{(1)}_{S_{dm} \leftarrow j}$ and $C^{(2)}_{S_{dm} \leftarrow j}$ to mean the sample histogram if S_{dm} had value j. Note that this step does not require a full recalculation of the sample histogram in

order to assess each possible new value: if the value S_{dm} in the samples is modified from j to j', we can simply decrease by 1 the entry corresponding to the old value ($C^{(1)}_{mj} = C^{(1)}_{mj} - 1$) and increase by 1 the entry corresponding to the new value ($C^{(1)}_{mj'} = C^{(1)}_{mj'} + 1$). For the 2-body histogram, we need to do this for all $M-1$ pairs of modes ($C^{(2)}_{mnjk} = C^{(2)}_{mnjk} - 1 \quad \forall n \neq m, \forall k$ and $C^{(2)}_{mnj'k} = C^{(2)}_{mnj'k} + 1 \quad \forall n \neq m, \forall k$) and their transpose. In this way we can quickly select the optimal value S^*_{dm} without actually changing the samples and without recomputing the sample histogram.

Algorithm 1 Produce a set of samples S

Require: M, D, $P^{(1)}$, $P^{(2)}$, cutoff ▷ num modes, num samples, target marginals, cutoff
$T^{(1)} \leftarrow \text{int}(DP^{(1)})$ ▷ target one-mode histogram initialized
$T^{(2)} \leftarrow \text{int}(DP^{(2)})$ ▷ target two-mode histogram initialized
$S \leftarrow \text{zeros}(D, M)$
for $m \in \{1, \dots, M\}$ **do**
    for $d \in \{1, \dots, D\}$ **do**
        sample $S_{dm} \sim P^{(1)}_m$
    end for
end for ▷ samples initialized
$C^{(1)} \leftarrow \text{zeros}(M, \text{cutoff})$
$C^{(2)} \leftarrow \text{zeros}(M, M, \text{cutoff}, \text{cutoff})$
for $m \in \{1, \dots, M\}$ **do**
    for $d \in \{1, \dots, D\}$ **do**
        $j \leftarrow S_{dm}$
        $C^{(1)}_{mj} \leftarrow C^{(1)}_{mj} + 1$
        for $n \in [1, M]$; $n \neq m$ **do**
            $k \leftarrow S_{dn}$
            $C^{(2)}_{mnjk} \leftarrow C^{(2)}_{mnjk} + 1$
        end for
    end for
end for ▷ sample histograms initialized
while convergence criterion not met **do**
    for $m \in \{1, \dots, M\}$ **do**
        for $d \in \{1, \dots, D\}$ **do**
            $S_{dm} \leftarrow S^*_{dm} = \text{argmin}_j \, ||C^{(1)}_{S_{dm} \leftarrow j} - T^{(1)}||_1 + ||C^{(2)}_{S_{dm} \leftarrow j} - T^{(2)}||_1$ ▷ greedy step
        end for
        randomly shuffle rows (along index d)
    end for
end while

---

1. 3rd order greedy

We have extended the greedy sampler to take into account 3rd order correlations using the 3-body marginal distributions across all triples of modes. We observe that the 3rd order sampler performs slightly worse than the 2nd order one described in the previous section. The main reason is that a large fraction of 3-body events have a probability that is too low to be picked up by the sampler. In the 3rd order sampler the target counts $T^{(3)} = \mathrm{int}(DP^{(3)})$ contain many values that are artificially zero as $0 < DP^{(3)} < 1$ even if D is $O(10^6)$. In order to show this effect we consider the first 12 modes from our system and a cutoff of 10. Figure S1 shows a histogram of the values of the 3-body joint probabilities that are below 10^{-6}. In comparison, the histogram of the probabilities of the 2-body events is in Fig. S2.

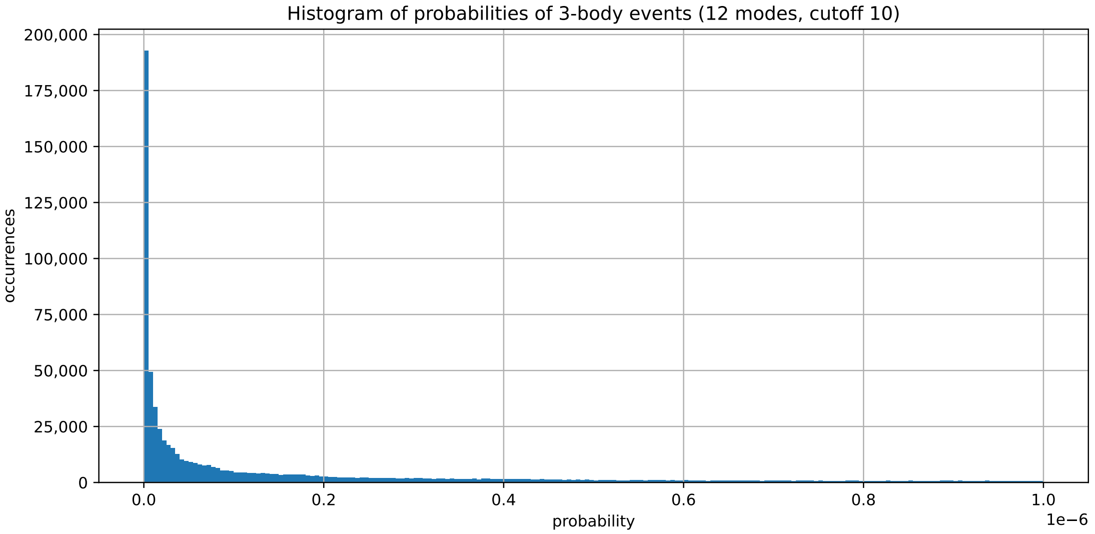

Figure S1. Too many 3-body events have a probability that is lower than $1/D$ (i.e. the “resolution” of the sampler). In this example with 12 modes and a cutoff of 10, if the sampler could work on optimizing 10^9 samples, almost 10% of events would still not be reproduced because events with probability below 10^{-9} will be treated as not occurring. For this reason the 3rd order greedy sampler is worse than the 2nd order one.

The histogram in Fig. S1 contains 53% of the total events (note that it spans only the 0-0.000001 range), i.e about half of the 3-body events have a probability that is below 10^{-6} and in fact 34% are below 10^{-7}, 18% are below 10^{-8} and 9% are below 10^{-9}, which means one needs to produce billions of samples in order for the sampler to consider more than 95% of the high-photon-number events. Compare this with the 2nd order case, where only 14% of the events has a probability lower than 10^{-6}, 4% lower than 10^{-7}, 0.5% lower than 10^{-8} and 0.05% lower

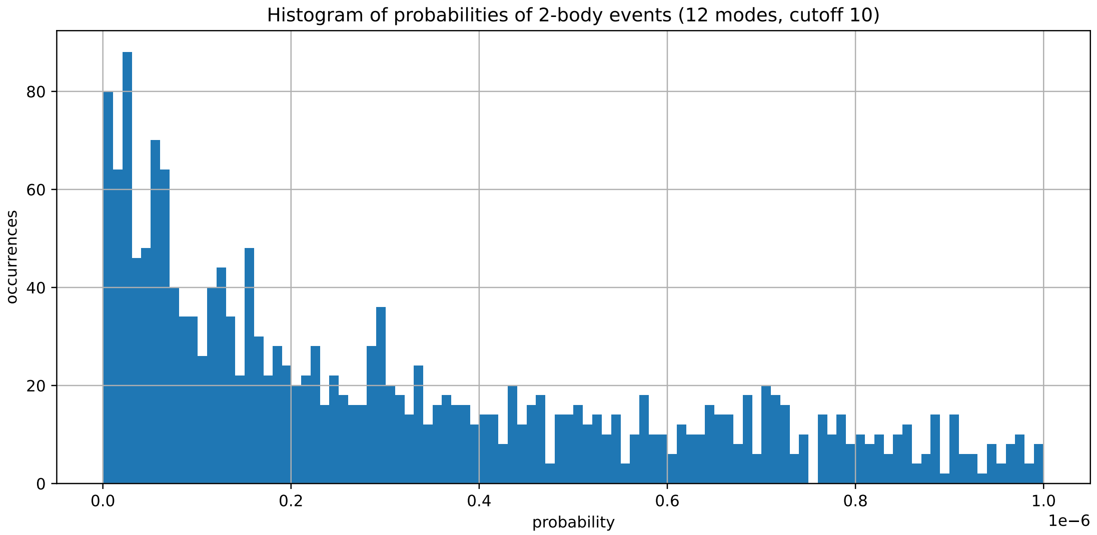

Figure S2. In the 2nd order case the sampler is able to take into account most of the 2-body events with a number of synthetic samples of $O(10^6)$.

Probability	10^{-6}	10^{-7}	10^{-8}	10^{-9}
3rd order	53%	34%	18%	9%
2nd order	14%	4%	0.5%	0.05%

Table S1. The percentage of events below a small probability threshold is significantly higher in the 3rd order case and leads to too many events being suppressed by the sampler.

than 10^{-9}. So in the 2nd order case, $O(10^6)$ samples contain most of the possible events. We summarize these numbers in table S1.

To illustrate the effect, we optimize 1 million samples for 12 modes and cutoff=10 over 5 sequential epochs with both the 2nd order and 3rd order sampler. For the 2nd order sampler the TVD contribution of the 1-body events is 0.0015 and of the 2-body events is 0.0373. For the 3rd order sampler, while the contribution of 3-body events decreases, the contributions from 1-body and 2-body events *increase* to 0.0020 and 0.0436 respectively, making it easier to determine whether the samples are synthetic.

Note that in the USTC experiment, which was performed using thresholded measurements, there are no "low probabilities" as any multi-photon event is clumped into the same click. In Fig. S3 we show the histogram for the same data, if we had used threshold detectors instead of PNRs. As can be seen, the lowest probabilities are significantly larger than those for the PNR data. In fact, no probability is too low.

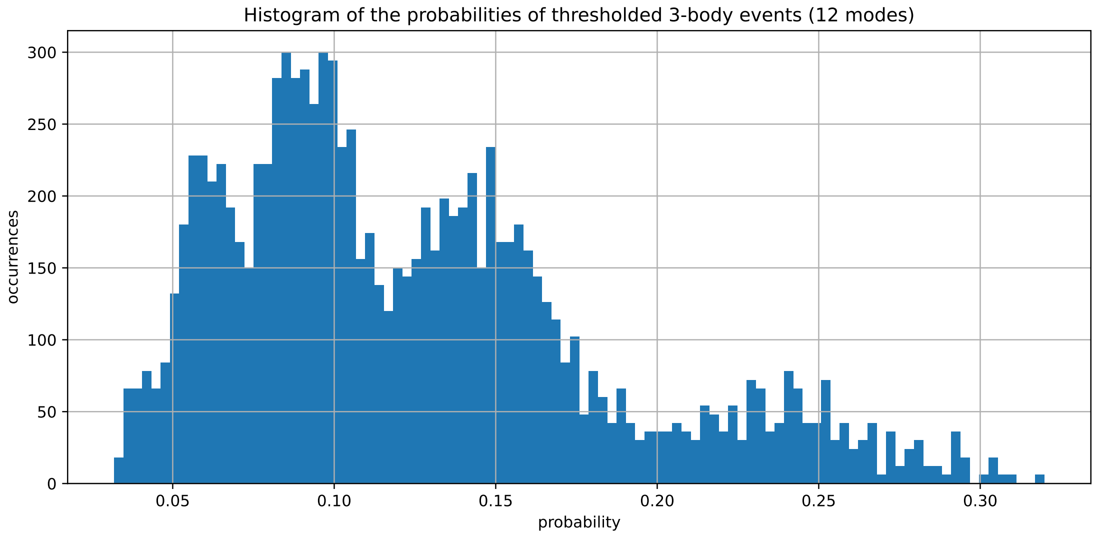

Figure S3. If the data is taken with threshold detectors rather than PNRs, there are no events with too low probability, so in this case a 3rd order greedy sampler is able to reproduce all the events.

1.2. TVD for classical adversaries in a 16-mode GBS instance

As explained in the main text, we run a GBS instance in the regime of few modes and low photon number, consisting only of 16 temporal modes instead of 216. Limiting the number of modes to 16 keeps the number of possible outcomes manageable which allows us to, firstly, compute the full probability distribution predicted by the ground truth and, secondly, compare it to the empirical probability distribution of the experimental data (see Fig. 2 in the main text). Comparing the probability distributions obtained by ground-truth simulations and experiment using the Bhattacharyya distance and total variation distance (TVD) gives us a good intuition of the fidelity of our experiment.

Here, we extend this analysis to all our classical adversaries. For that purpose, we create synthetic mockup samples for each spoofer and sample the probability distribution of possible 16-mode outcomes in the same way as we are doing for the experimental samples. Here we make sure to use the same number of samples for all candidates at each N, so none of the adversaries is given a statistical advantage over the others. Figure S4 illustrates the TVD as a function of total photon number N between the ideal theoretical distribution and the distributions sampled from the respective adversaries.

In addition to the classical adversaries, we also generate samples directly from the ground-truth probability distribution to illustrate the optimal bound and the effect of finite samples.

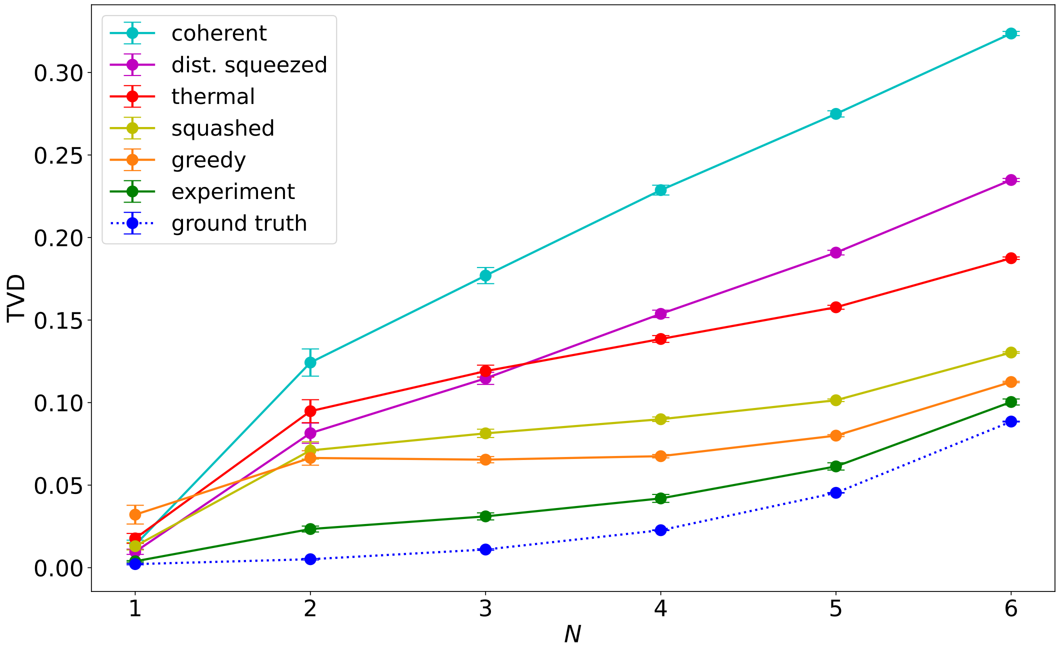

Figure S4. **Total variation distance (TVD) of the 16-mode GBS instance as a function of total photon number** N. The samples generated from the ground truth (dotted blue) are a full simulation of the 16-mode state and hence yield the optimal TVD given the finite number of samples which, for each N, is set to be equal to that of the experiment and all adversaries. For all photon numbers up to $N = 6$, the experiment is closer to the ground truth than the adversarial distributions corresponding to coherent, distinguishable squeezed, thermal, and squashed states and the greedy sampler, respectively. The error bars on the experimental data points (green) are given by the standard deviation of the nine repetitions of the 16-mode state within each acquisition cycle. The other error bars are the standard errors of the sampled TVDs.

Coming from the ground truth directly, these samples are ideal and the TVD of their sampled distribution is only lower-bounded by statistical error: the higher N, the more possible outcomes there are, the less accurately the probability of each outcome can be sampled. The TVD of these ideal samples and their statistical error is illustrated by the dotted blue line in Fig. S4.

Note that in order to achieve 8.3×10^7 experimental samples we run a total of 9.3×10^6 acquisition cycles with nine repetitions of the 16-mode state in each cycle. The modes of these nine repetitions experience the same interferometer but end up in different combinations of PNR-detector channels which is why each repetition has its own, slightly different, ground truth. This subtlety is not taken into account in Fig. 2 on the main text where, for the sake of quantity, we bunch the 8.3×10^7 samples together and compare them against a single ground truth. It

is, however, taken into account in Fig. S4 where making 8.3×10^7 synthetic samples for each spoofer is too expensive anyway. Here, we compare each repetition of the experimental samples with its own ground truth which leads to slightly different TVD values compared to the ones reported in the main text.

We now briefly investigate the marginalized TVD method considered in Ref. [9] using the 16-mode GBS instance. The results of this, presented in Fig. S5, show that the greedy spoofer typically has a smaller TVD to the ground truth than the experiment when heavily marginalized. However, as the number of modes we consider starts to approach all the modes of the system (i.e., as we marginalize over fewer modes) the experiment outperforms the spoofer in this metric. We cannot reproduce this exact analysis to directly verify that this trend holds for the 216-mode instance, since the computational resources and number of samples required to estimate probabilities suffers an exponential blow up. On the 216-mode data, for heavy marginalization with few modes retained, and small photon number cutoffs, the greedy sampler does produce samples lying slightly closer to the ground truth than do the experimental samples; this was confirmed for up to 17 modes retained with cutoff 2, 10 modes retained with cutoff 3, and 8 modes retained with cutoff 4. Yet, this smaller-scale 16-mode GBS instance, which allows us to avoid truncation of photon number and for which we can reliably estimate probabilities with a reasonable number of samples, serves as a strong counterexample to the proposition that the TVD metric, under heavy marginalization, has any conclusive bearing on the performance of the exact same experiment when the full outcome space over all modes is retained.

1.3. Numerical specifications of the classical adversaries

For the cross entropy score illustrated in Fig. 3a of the main part, we generated one million synthetic samples for each classical adversary, respectively. They were constructed under the condition to match the mean photon number of each mode to the ground truth (as verified by the low total variation distance (TVD) of the first-order cumulants in Table S2). Moreover, the table shows that our greedy algorithm successfully produced samples that even match the *second*-order cumulants better to the ground truth than the actual experiment does.

For the bayesian score illustrated in Fig. 3b of the main text, the classical adversaries were constructed by applying the ground-truth transfer matrix to the respective optical input states. These Gaussian adversaries were built under the imposed condition of a perfect overlap with

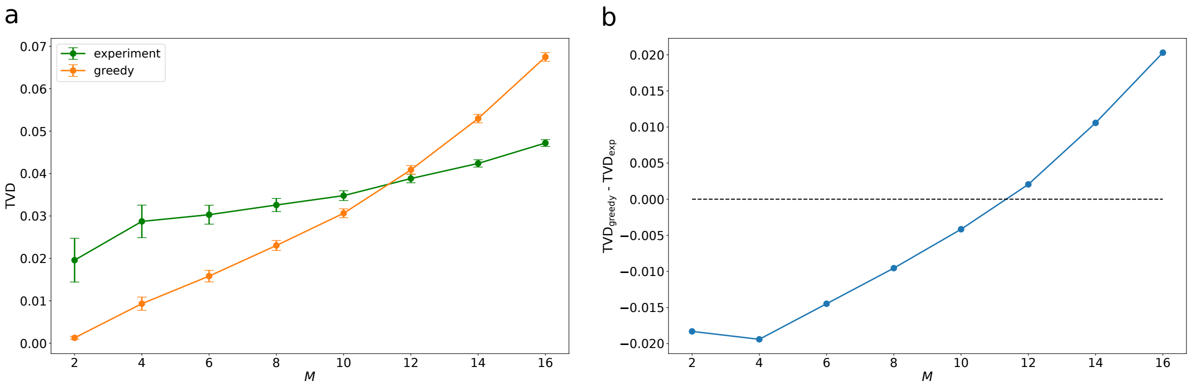

Figure S5. **Marginalized TVD of the 16-mode GBS instance for the four-photon subspace**. **a** TVD as a function of the modes retained (M) of the experiment (green) and the greedy sampler (orange) to the ground truth. For both, all four-photon events from a 10 million sample data set are used. Each point corresponds to the average of 100 randomly selected subsets of modes, except for $M = 16$ for which there is only one set. The error bars correspond to the mean standard error of the individual TVDs. **b**, TVD difference as a function of the number of modes retained (M).

Table S2. Total variation distance (TVD) for first- and second-order cumulants of experimental samples and classical adversaries vs. the ground truth. For the cross entropy score found in the main text, the cumulants were obtained by the mean photon number and photon-number covariance of the (experimental and synthetic) samples. For the bayesian score, the cumulants of the classical adversaries were obtained from their covariance matrices. Here, $\langle N \rangle$ corresponds to the experimentally measured mean total photon number and r is the average squeezing.

					TVD to ground-truth cumulants	
Score	Modes	$\langle N \rangle$	r	Candidate	1st order $[10^{-3}]$	2nd order
Cross entropy	216	21.120	0.533	Experimental	4.613	0.0759
				Squashed	1.283	0.228
				Thermal	1.249	0.305
				Coherent	1.267	0.310
				Dist. squeezed	1.313	0.153
				Greedy	0.050	0.0716
Bayesian	72	22.390	0.886	Experimental	2.355	0.0366
				Squashed	0	0.119
				Thermal	0	0.297
				Dist. squeezed	0	0.205

the first-order cumulants of the ground truth, which is verified by their TVD of exactly zero.

2. EXPERIMENTAL SETUP AND METHODS

2.1. Squeezed-light source

A detailed schematic of the setup surrounding the squeezed-light source is shown in Fig. S6. In what follows, we describe the optical parametric oscillator (OPO) cavity geometry and spectral properties, how the pump pulses and the lock beam for the OPO are generated.

We use a hemilithic cavity geometry [10] to generate squeezed states of light. This OPO is formed as a semi-monolithic doubly-resonant cavity around a 10 mm periodically poled KTP (PPKTP) crystal and a piezo-mounted mirror as shown in Fig. S6. The fundamental wavelength is 1550 nm, while the pump is at 775 nm. Given the Type-0 phase matching of the PPKTP crystal, the pump and the squeezed-light beam share the same polarization, and by controlling the temperature of the crystal, one can reach simultaneous resonance for both wavelengths. To achieve double resonance and near single-mode operation, we selected appropriate coatings on both the crystal surfaces and the output coupler as depicted in Fig. S7.

The pump light consists of a 6 MHz stream of 3-ns-duration rectangular pulses with an average power of 3.7 mW at 775 nm. The power was chosen to ensure sufficient brightness, and the duration chosen to achieve single-temporal-mode operation [11]. As depicted in Fig. S6, it originated from the main ultra-low phase noise fiber laser, centered at 1550 nm. A 4 GHz lithium niobate electro-optic intensity modulator (pulse generator) carves the pulses, under the control of an arbitrary waveform generator (digital pulse control). Those pulses are then amplified using an Erbium-Doped Fiber Amplifier (EDFA) and upconverted to 775 nm via second-harmonic generation (SHG) using a fiber coupled MgO:LN ridge waveguide. The resulting train of pump pulses is then directed towards the OPO. The average power of the pump light can drift over timescales of minutes and hours. To overcome this power drift during longer sequences of measurements, we actively stabilize the pump intensity using an acousto-optic modulator (AOM) before the OPO, while monitoring pump light transmitted through the OPO using a photodiode (PD_2).

The train of single-mode squeezed vacuum pulses emitted from the OPO is separated from the pump at a dichroic mirror (DM), coupled into a single-mode fiber, and directed towards the fully programmable photonic processor.

To continuously lock the OPO cavity, using the Pound–Drever–Hall (PDH) technique, we

prepare the 'OPO lock light' beam such that i) it is not at the same wavelength as the squeezed pulses, ii) it has an orthogonal polarization to the pump pulses (to prevent continuous generation of squeezed light), and iii) it is independent of the pump pulses. The preparation of the OPO lock light, depicted by a solid green line in Fig.S6, is as follows. Part of the main laser light is modulated at ≈ 7 GHz to generate side-bands, by means of a phase modulator. The resulting light is passed through an etalon cavity (with a linewidth of 2 GHz and FSR of 60 GHz) to filter out everything except the $+1^{st}$-order side-band. We monitor the spectrum using an optical spectrum analyser (OSA). It is then amplified and phase modulated with a second phase modulator, driven by a RedPitaya field-programmable gate array (FPGA) board, to generate side bands at 40 MHz for the PDH lock. Subsequently, the light passes through a temperature-controlled fiber-coupled MgO:LN ridge waveguide (SHG) which up-converts ≈ 1550 nm light to ≈ 775 nm. The transmitted light through the OPO is detected by a photodiode (PD1), from which the PDH error signal is derived.

2.2. Loop-based programmable interferometer

Trains of single-mode squeezed vacuum pulses from the source are directed towards the fully programmable photonic processor, consisting of three loop-based interferometers in series. Each loop $\ell = 0, 1, 2$ is characterised by a variable beamsplitter (VBS_l) and a fiber delay line (τ, 6τ and 36τ), where $\tau \approx 167$ ns.

Each VBS is decomposed into a programmable phase shifter and a programmable Pockels cell as shown in Fig.S8. In more detail, the Pockels cell system consists of a variable amplifier (±600 V) combined with a non-resonant electro-optic (EO) Pockels cell allowing fast, arbitrary polarization rotation. The repetition rate of 6 MHz is synchronized with the arrival time of incoming squeezed-light pulse trains. The EO crystal, a 2x2 mm^2 Rubidium Titanyl Phosphate (RTP) crystal with optical transmittance ≥ 99% at 1550 nm, is sandwiched between two polarizing beamsplitters and has a quarter-wave plate in front [12]. A remotely controlled arbitrary function generator applies $\tau = 167$ ns wide square pulse sequences of variable amplitudes to the modulator, introducing a local polarization rotation of mode k leading to variable transmittance $t_k^\ell \in [0, 1]$ for loop $\ell \in \{0, 1, 2\}$. In contrast, a squeezed light pulse is 10 ns wide, well within the 167 ns gate length. The external phase gate, located in front of each loop-interferometer, is controlled using a similar system: an amplifier (±300 V), remotely controlled by a function

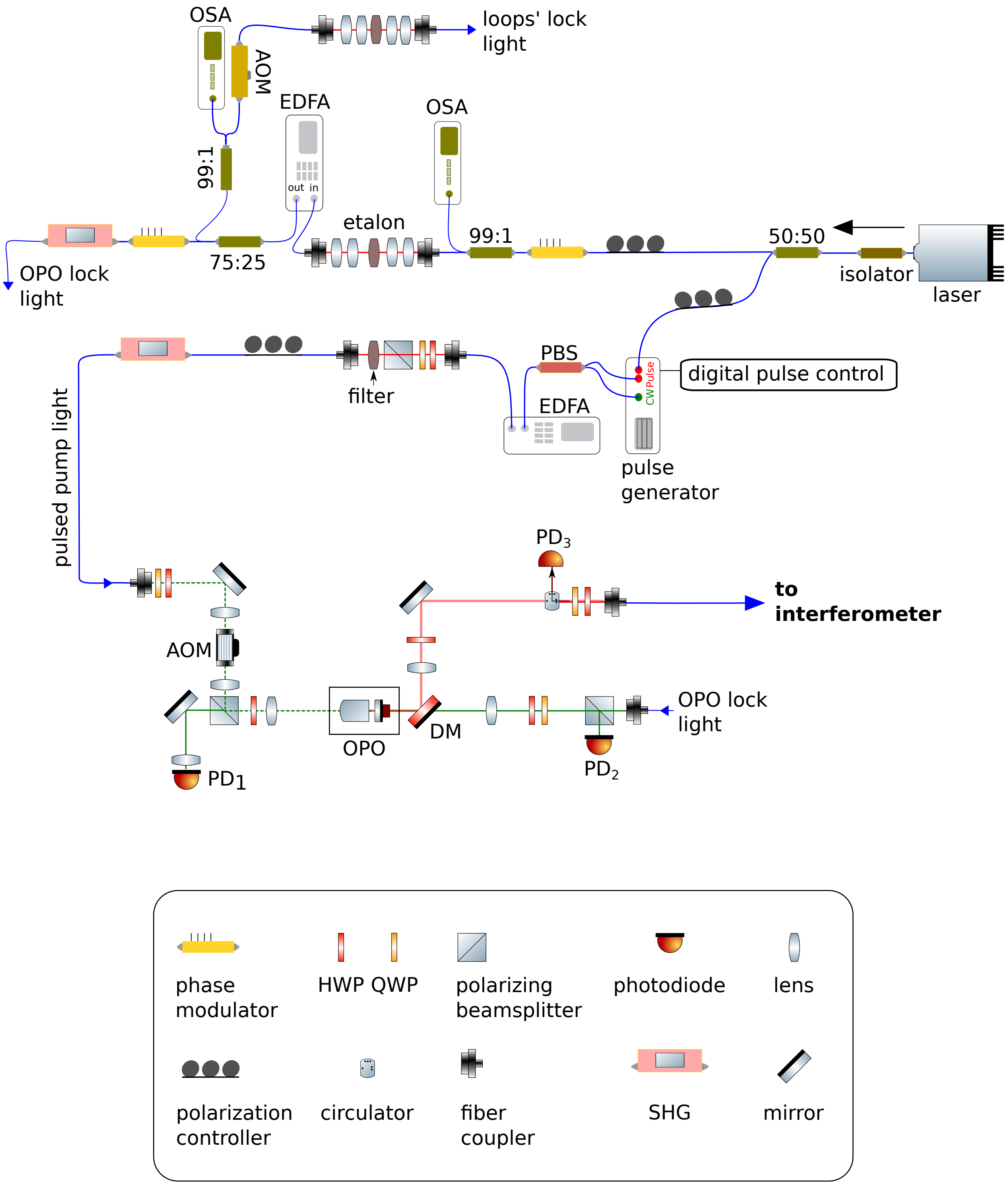

Figure S6. **Squeezed-light source**. OSA; optical spectrum analyser, EDFA; erbium-doped fibre amplifier, SHG; second-harmonic generation, OPO; optical parametric oscillator, AOM; acousto-optic modulator, DM; dichroic mirror, HWP; half-wave plate, QWP; quarter-wave plate. PD_1, PD_2, and PD_3 are used for, respectively, OPO lock light, pump power stabilization, and the loops' lock light. Dashed green and solid lines represent pulsed pump light and OPO lock light, respectively, whereas transparent red line depicts the generated squeezed light. Blue lines/curves depict single-mode fibers.

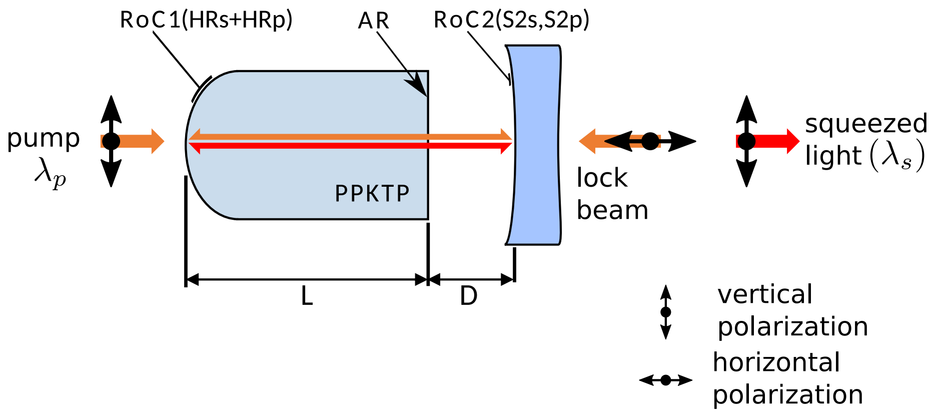

Parameter	Value
λ_p	775 nm
λ_s	1550 nm
RoC1	12 mm
HRs	99.9%
HRp	90%
AR	<0.2%
RoC2	25 mm
S2s	90%
S2p	90%
L	10 mm (fixed)
D	22 mm (variable)

Wavelength	Waist (μm)	FSR (GHz)	FWHM (MHz)	finesse
1550 nm	40 @ D $\perp$ 4.6 mm	3.7	66	57
775 nm	28 @ D $\perp$ 4.6 mm	3.7	127	29

Figure S7. **OPO Hemilith cavity geometry and spectral properties**. PPKTP; Periodically poled KTP crystal, λ_p; pump wavelength (775 nm), λ_s; squeezed-light wavelength (1550 nm), RoC; radius of curvature, $HR_{s(p)}$; high-reflectivity coating at 1550 nm (s) and 775 nm (p), AR; anti-reflection coating, FSR; free spectral range, FWHM; full-width at half maximum.

generator, modulates a non-resonant EO phase shifter (Lithium niobate crystal, ≥ 99% optical transmittance), for arbitrary phase adjustments of linearly polarized input squeezed-light pulses. In our experiment the amplifier driving this component was limited to a voltage range yielding $-\pi/2$ to $\pi/2$ phase, but increasing the output range of the amplifier allows the full range of $-\pi$ to π to be programmed. Both the phase shifter and Pockels cell systems are manufactured by QUBIG GmbH.

In order to achieve high in-coupling efficiency to the loop delays (approaching 97% on average) throughout the entire interferometer, we use 4f or 8f imaging systems by means of spherical lenses and graded-index (GRIN) lens fiber couplers. The loss introduced by the delay line in each loop is measured to be 5% for the first, and 7% and 12% for the second and third loops, respectively. These losses are due to free-space-to-fiber coupling efficiency and the delay line fibers' intrinsic loss. To match the relative lengths of each loop delay and to optimize the temporal overlap between different time bins (to the accuracy of 250 ps), we use two spherical lenses, placed at the output of each delay line. By adjusting the position of the lenses and fiber coupler, we can optimize the round-trip time through each loop using interference between two pulses.

According to the manufacturer of the fiber, dispersion at our wavelength (1550 nm) is < 18.0 ps/(nm*km). The line-width of our OPO is ~ 66 MHz, corresponding to ~ 0.0005 nm. Given

a train of 216 pulses, the delay between the first (going through fiber) and the last pulse (free-space) would be 215 $*$ (34 m) = 7.3 km. Note that this length of fiber is 5.9 times longer than our longest coil and is used only to derive an upper bound on the amount of dispersion expected. With this propagation length, we can estimate the time spreading experienced by the first pulse of approx. < (18 ps) $*$ (0.0005 nm) $*$ (7.3 km) = 0.0657 ps, or < 65.7 femtoseconds. This spread is at least six orders of magnitude smaller than the original pulse length, and thus, to very good approximation, dispersion will not introduce significant distinguishability between long-range pulses in our experiment.

Other concerns could be raised about polarization mode dispersion (PMD). According to the same manufacturer of the fiber, the reported value of PMD is <0.04 ps/sqrt(km) for concatenated lengths of fiber. Using the same propagation length as described in the previous paragraph, we can estimate the temporal broadening experienced by one pulse to be <(0.04 ps)*sqrt(7.3 km) = 0.11 ps, or < 110 femtoseconds. This spread would also be approximately six orders of magnitude smaller than the original pulse length, and thus, to very good approximation, PMD will not introduce significant distinguishability between long-range pulses.

Finally, at the output of the interferometer, we filter out additional non-degenerate two-mode squeezed states from the OPO, in the $\pm 1, \pm 2, \ldots$ FSR modes. For this purpose, we use a pair of fibre Bragg gratings (FBGs). The FBGs have a flat top reflection band of width approximately 5 GHz (corresponding to 0.04 nm @ 0.5dB) and a relative offset of approximately 1 GHz. The first FBG is used in reflection. The frequency of the squeezed light is tuned so that nothing but the degenerate frequency band and the +1 FSR side-band is reflected. The second FBG is used in transmission and reflects the +1 FSR mode while transmitting the squeezed light. Combining these filters suppresses all the other OPO modes by more than 30 dB. In addition, we employ a broadband dielectric filter with a bandpass of 10 nm around 1550 nm and around 60 dB suppression of room and pump light. These filters in conjunction with fiber couplings have a total transmittance of around 80% for a single-mode squeezed pulses.

1. *Implementation errors of gate parameters*

In this section, we discuss the expected errors in the implementation of beamsplitter and phase shifter parameters. We then simulate the impact of those errors on the second-order cumulants. Prior to the experiment, we have characterized the system performance of all three

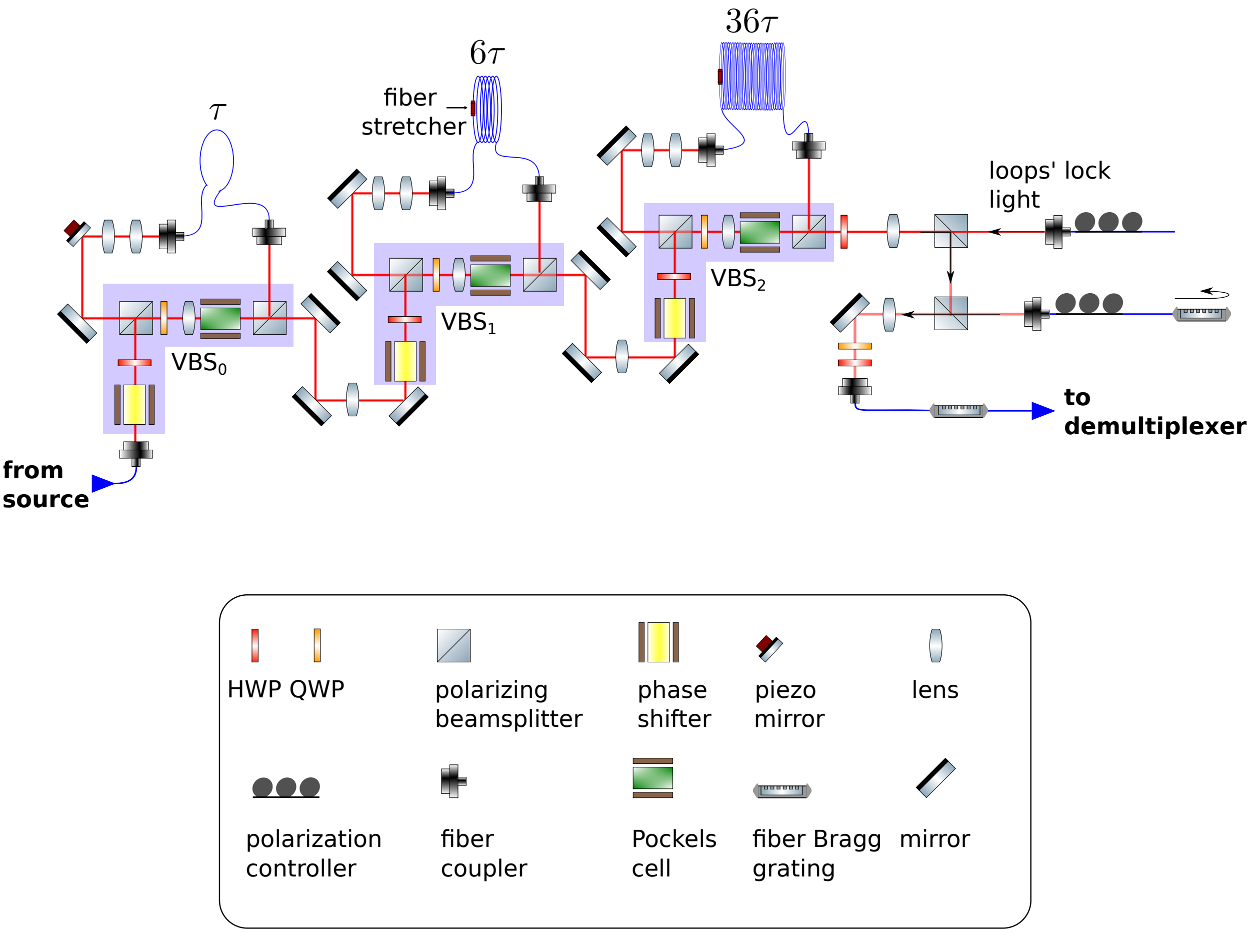

Figure S8. **Loop-based programmable interferometer**. VBS stands for variable beamsplitter, highlighted by purple area for each loop. See details in text.

phase shifters and three amplitude modulators (beamsplitters) using coherent states and repeated sequences of gate parameters. By comparing the expected amplitudes with measurements, we found a deviation of less than 1% on average for all devices, while using a step width of 167 ns and analyzing a temporal window of 25 ns where the squeezed light pulse is meant to pass the gate. We also quantified the shot-to-shot repeatability (reproducibility) of the devices by analysing a repeated sequence of random amplitudes several times, and found typical errors to be less than ±0.8%.

In Fig. S9, we apply a range of amplitude and phase errors to our theoretical models. Amplitude noise imposed by the variable beamsplitters, and phase noise imposed by the phase shifters are investigated using Monte-Carlo simulations of a Gaussian noise with standard deviation σ_T and σ_ϕ, respectively. In addition to random noise, we investigate the effect of constant amplitude offsets caused by possible improper alignment or calibration of the variable

beamsplitters. (Constant offsets in the phases applied by our phase shifters will not affect the photon-number distribution and are therefore neglected in this analysis.) In order to get an intuition on the impact of these errors on the photon number distribution, we build the difference of the second-order cumulants that are simulated under addition of gate errors to the experimentally observed ones (blue lines). The red vertical line corresponds to the noise regime where we estimate our devices to be from prior calibrations, situated where the cumulants' noise response is more or less flat in all cases.

In order to obtain an upper bound on the combined imperfections of our modulators, we also calculate the difference between the cumulants, simulated under different error assumptions, and the ground truth as a function of the respective gate imperfections (orange lines). The intersection of these orange lines with the horizontal dashed line (the mismatch between experiment and ground truth) marks an upper bound to implementation errors of gate parameters: For instance, if the *entire* mismatch between experiment and ground truth would be accounted to nothing else but amplitude errors of the beamsplitters, these fluctuations would have a standard deviation of ~ 0.055. In a similar fashion, we obtain an upper bound of $\sigma_\phi \sim 0.025\pi$ for the phase shifters and an upper bound of $\Delta_T \sim 0.08$ for the beamsplitter offsets. In the actual experiment, of course, several small imperfections act together and we thus expect the modulators to be significantly better, close to specified levels marked by the red vertical lines.

2. *Phase stabilization of the interferometers*

The main challenge in locking the interferometers is the combination of different requirements, in particular: PNR detector compatibility, introducing minimum extra loss on the squeezed light, and independent phase-stabilization of the three loops.

In order to make the lock beam PNR compatible we use a 1550 nm beam, 7 GHz frequency-shifted with respect to the squeezed light, generated together with the OPO lock light. It is injected counter-propagating compared to the squeezed light (i.e. propagating from the third loop towards the first loop) on a 99.5 % beamsplitter and coupled out on a free-space circulator (5 % loss to the squeezed light) after the three interferometers with 1μW of power. About 1 part in 1000 of the lock beam is back-reflected from the interferometer and travels towards the PNRs. The 7 GHz frequency shift is tuned such that the beam is transmitted through the first

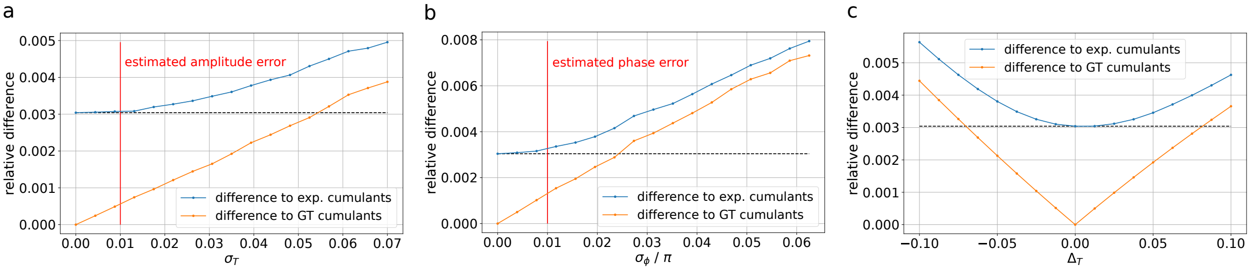

Figure S9. **Implementation errors of gate parameters**. **a**, Amplitude noise of the beamsplitters: Relative difference of simulated and experimental second-order cumulants with respect to the standard deviation of the three beamsplitters' amplitude noise (σ_T). For each σ_T, we perform a simulation of the GBS instance used in Fig. 4 of the main text. These simulations yield the second moment of the photon-number distribution which are compared against the moments obtained from the experiment (blue) and the ones of the ground truth (orange). The red vertical lines correspond to the noise levels obtained from independent calibration measurements (which are also verified by the manufacturer's specifications). The dashed horizontal line marks the difference between the experimental statistics and the ground truth. The y-axis is computed by the average of $2|cov_0^{i,i+d} - cov_{\mathrm{sim}}^{i,i+d}|/(cov_0^{i,i} + cov_0^{i+d,i+d})$ where cov is a photon-number covariance matrix, i runs from 0 to 215, and $d \in \{1, 6, 36\}$. **b**, Phase shifter noise: Same as (**a**), except we simulate phase noise of the phase shifters instead of the amplitude noise of the beamsplitters. (**c**) Beamsplitter offsets: Same as previous, with constant transmission offsets added to the beamsplitters.

fiber Bragg grating (which reflects the squeezed light) and reflected on the second fiber Bragg grating (which transmits the squeezed light), giving an additional 50 dB attenuation. Although such a well-attenuated back-reflected lock light does not saturate or heat up the PNRs more than the squeezed light, it would still be a significant source of stray counts. Therefore, the lock beam is gated using an AOM, so it is only present *between* pulse trains, each containing 216 modes for example. In other words, the loops' lock light and the squeezed modes enter the interferometers alternating with a time window of 65 μs for the former and 35 μs for the latter and thus, a clock rate of 10 kHz.

To make the loop-interferometers' locks independent from one another while only using a single lock beam, we generate the error signals in each interferometer by an amplitude modulation that is minimally affected by the other loops. In more detail, the beamsplitter is set to couple all light into the delay line (*cross* state) and applies a modulation of 1.3 MHz, 3.5 MHz, and 1.8 MHz for loop 1, 2 and 3, respectively, with a depth of around $V_{\pi/2}/10$. For the unmodulated component of the light (the carrier) the loop is in the cross state, and therefore it does not interfere. The modulated side band – for which the beamsplitter deviates from the cross state – interferes with itself, creating the error signal. With each loop having a different frequency, the

three error signals are decoupled from one another.

After the circulator, the light is detected on a high-gain 5 MHz photodetector. The electric signal is high-passed, amplified and split in three signals. Each part is mixed down with the frequency of the respective loop and low-passed in order to form a lock-in measurement. Each of these signals is then sent to a Red Pitaya running PyRPL[13], which enables proportional–integral feedback. The feedback is sent to high voltage amplifiers connected to a piezo-mounted mirror in the first and to fiber stretching piezos in the second and third loops. Finally, to decrease the phase shifts from thermal drifts we place each fiber-delay coil in a temperature-stabilized polystyrene box.

2.3. 1-to-16 demultiplexer

As mentioned in the main text, to bridge the gap between the repetition rate of the squeezed modes (6 MHz) and the inverse relaxation time of our photon-number resolving (PNR) detectors, a 1-to-16 demultiplexer (demux) is assembled. A detailed schematic of the demux is shown in Fig. S10. The demux effectively slows down the repetition rate of the squeezed modes from the initial 6 MHz to the final 375 kHz for each PNR detector channel. To achieve this, there are 15 low-loss resonant electro-optic polarisation modulators (EOMs), from QUBIG GmbH, grouped in four different layers. EOMs in each layer have a preset frequency: one at 3 MHz, two at 1.5 MHz, four at 750 kHz and eight at 375 kHz. Each EOM is sandwiched between two polarizing beamsplitter (PBS) and a quarter-waveplate at 45 degrees in front. The modulators are driven by a standalone unit (also from QUBIG GmbH), generating multiple phase locked sine wave signals temporally synchronized with the input train. The switching extinction ratio is measured to be above 200:1 on average for all modulators. The average total transmittance of the demux is over 90%, including 95% free-space-to-fiber coupling efficiency of each output, achieved using a pair of lenses to image efficiently the input to each output.

2.4. Photon number resolving detection

A transition-edge sensor makes use of the dynamic phase transition from the superconducting to the normal state of a thin film. In this application the device includes 20 x 20 μm square of tungsten film, Niobium leads and bond pads as well as a dielectric mirror optimized for

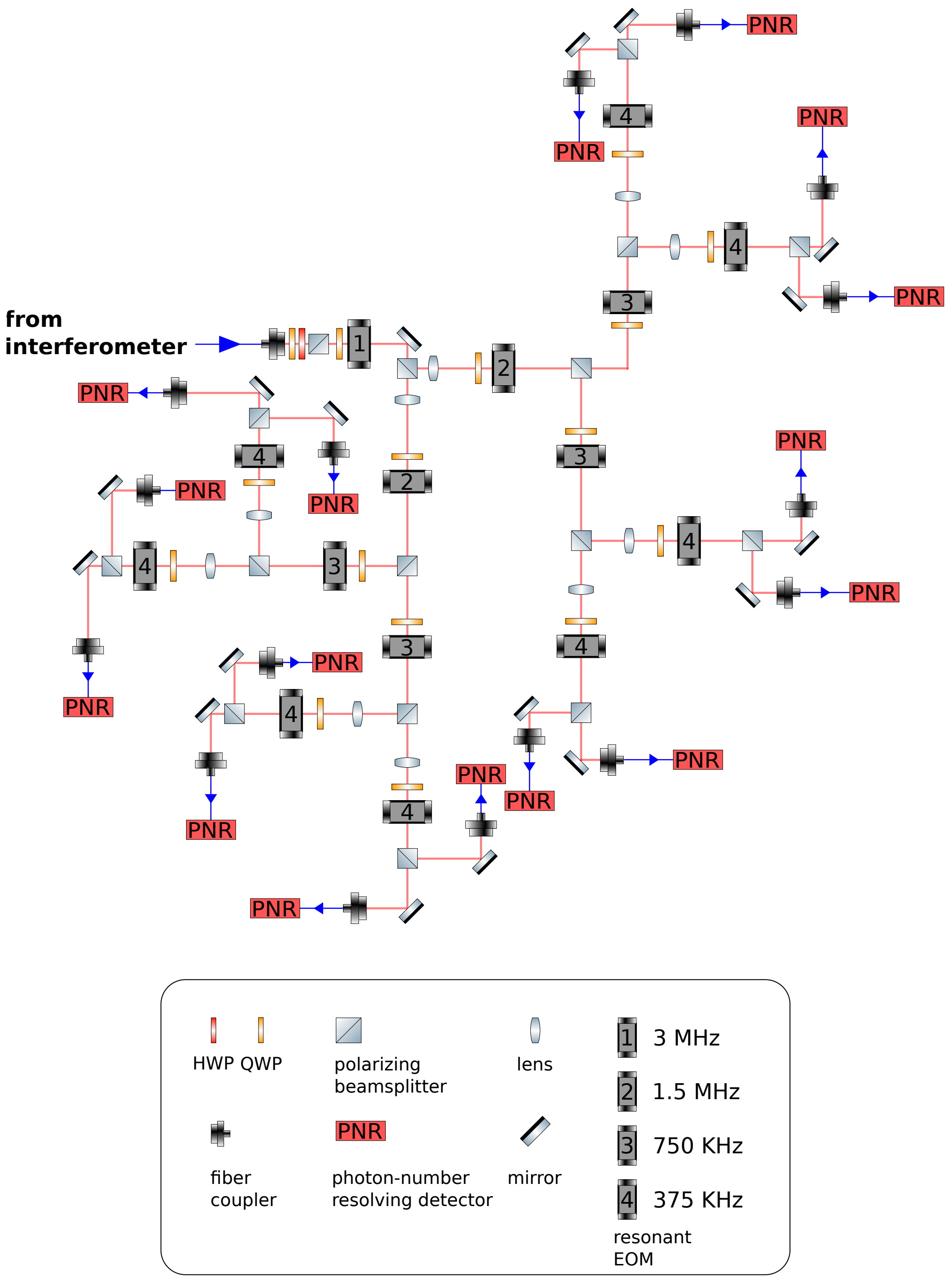

Figure S10. **1-to-16 demultiplexer**. See text for description

1550 nm between the tungsten and the silicon substrate [14]. The substrate is cooled well below the film's superconducting transition temperature and the detector is voltage-biased within the transition, with Joule heating electro-thermal feedback stabilizing the device. When photons are absorbed by the detector the temperature, and thus the resistance, increases momentarily and this change can be read out by a change in current through the input coil of a DC-SQUID

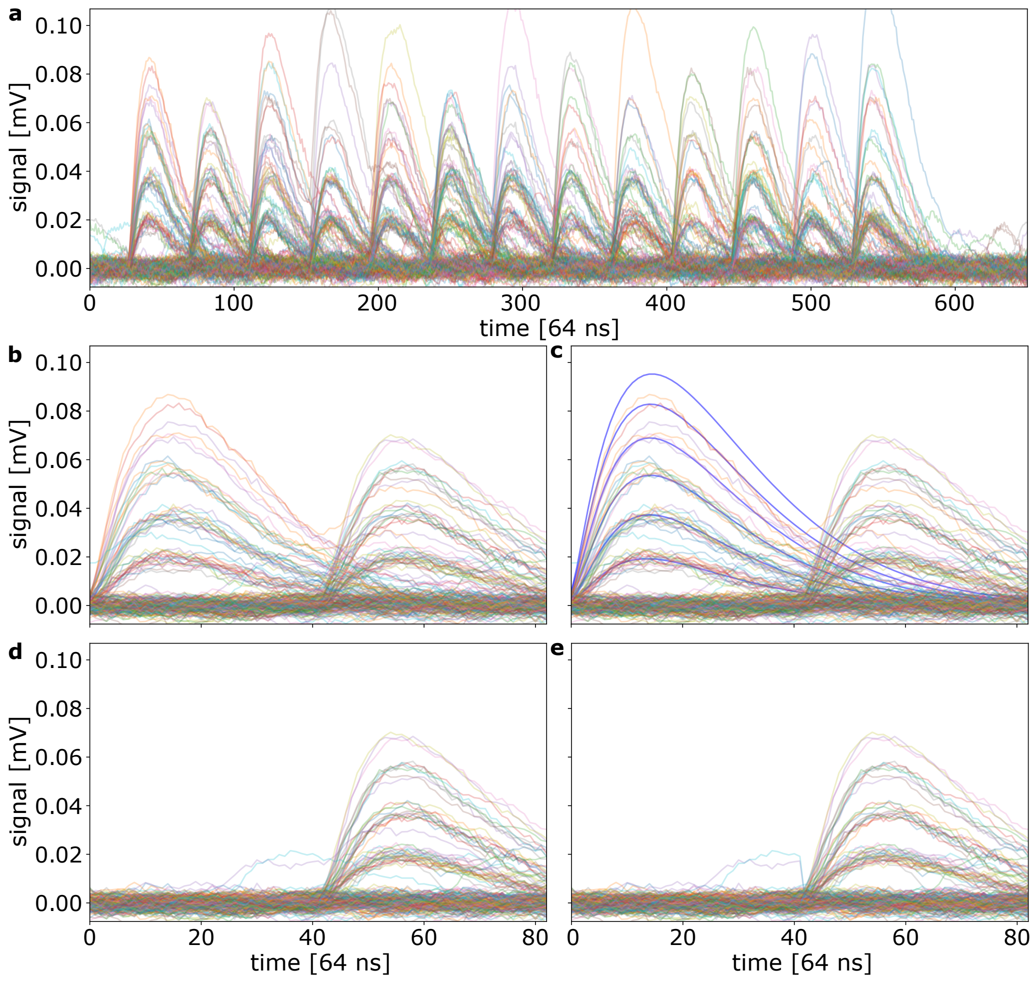

Figure S11. **PNR data and tail subtraction**. **a,** 200 repetitions of 13 modes, separated by 1/375 kHz, as measured in one PNR channel of the demux, with 216 squeezed pulses at its input. **b,** Zoom in on the first two pulses from panel **a**; photon numbers of up to 5 are observed. **c,** Same as in panel **b**, including the relevant calibrated shapes overlayed. Each waveform in pulse 1 will be assigned the photon number to whose template it has the smallest distance as described in the text. **d,** The calibrated shapes corresponding to each identified photon number subtracted, so that pulse 1 does not interfere with pulse 2. **e,** two stray events have been identified and their tails also subtracted. Pulse 2 is now ready for photon-number extraction.

array.

The output voltage signal from the SQUIDs is amplified and digitized at 15.625 MHz giving 42 points per pulse from which the photon number is extracted. For a 216 mode GBS state, each PNR will receive 13 or 14 pulses. As a first step in the analysis, an offset is subtracted. The output data from 200 repetitions of such a pulse train is shown in Fig. S11**a**.

As described in the Methods section of the main text, to extract the photon numbers from a pulse train, we first calibrate the individual detectors to obtain the mean unique shape of

each photon number using well separated pulses and the area-based photon number extraction approach [15]. The unique shapes are extracted once and reused as long as the detectors are unchanged.

The main procedure to extract photon numbers from the pulse train is to calculate the distance between the data and each unique shape and then assign the photon number to the least distance. Fig. S11**c** shows the unique shapes overlaid with the data for pulse 1 and 2 of the pulse train. The distance is only calculated within the 42 points and weighted by the unique shape of the single-photon trace.

As seen in Fig. S11**b**, the pulse shapes of pulse 1 extends into pulse 2. To minimize the effect of crosstalk between sequential pulses, we subtract the tails of the unique shape from the data as shown in Fig. S11**d**. In this way, correctly-identified photon numbers with no stray event will look like 0 events (vacuum) after subtraction.

In the example given by Fig. S11**d**, the waveform of two stray events can be seen. These can occur due to room light, back reflections or finite extinction in the demux or loop. These stray events are identified using the points right before the pulse arrives. If the mean of the waveform is more than four standard deviations above the one of the zero-photon trace, a tail normalized to the height is subtracted. This procedure was also performed on the first pulse in Fig. S11**b** which further reduces miscounts caused by stray events. The process can then be repeated for the next pulse and so forth. The stray-event rate from all lock beams and room light amounts to an average of 0.003 photons per pulse .

2.5. System transmittance

As described in Section IV.E in the main text, the common loss as well as the loss imposed by the loops and the 16 demux/PNR channels, respectively, are extracted from a systematic calibration routine, and the respective transmittances are reported in Table S3.

2.6. Generation and evaluation of two-mode squeezed states

As mentioned in Section IV.E of the main text, we synthesize two-mode squeezed states in order to obtain crucial information about our experiment, including the single-modeness of our squeezed-light source, the intrinsic phase offsets in each loop, and the optical loss in the

Table S3. Transmittance values obtained from the calibration routine. The common transmittance describes the minimum amount of loss that all modes share. It corresponds to the transmittance experienced by a mode that has bypassed all delay lines and ends up in our most efficient demux/detector channel. The transmittance value of each loop corresponds to a roundtrip in the delay line, followed by a second transit through the VBS optics. The demux/detector transmittances are actually described by a list of 16 values, normalized by the most efficient channel. In this table we report the average of these values. The number in parentheses corresponds to the uncertainty of the last digit displayed.

Element	η
Common	0.482(9)
1τ loop	0.918(9)
6τ loop	0.889(5)
36τ loop	0.850(5)
Average demux/detector channel (relative)	0.930(3)

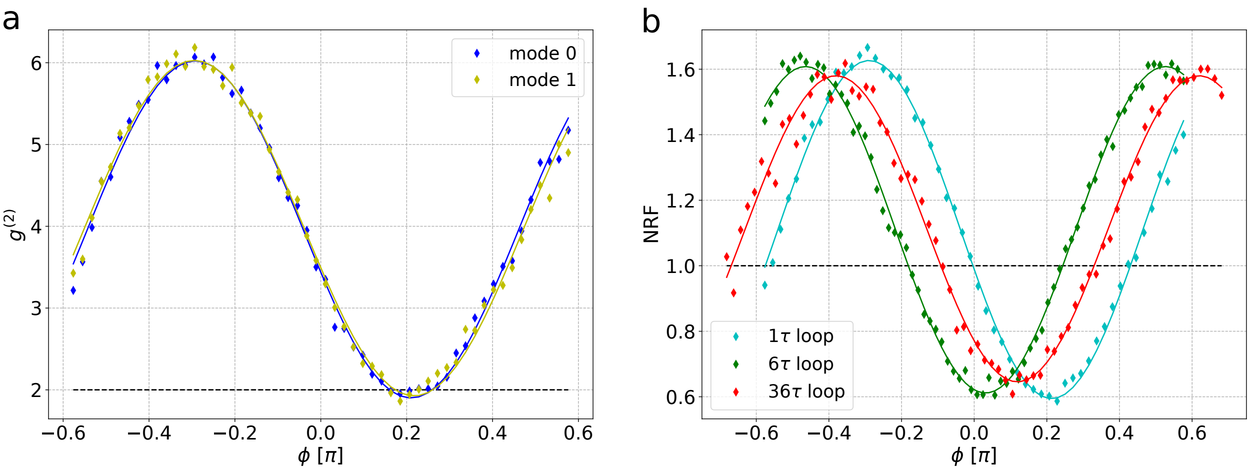

Figure S12. **Photon-number statistics of two-mode squeezed states**. Panel **a** illustrates the unheralded second-order correlation $g^{(2)}$ as a function of the applied phase ϕ using phase gate and beam-splitter assigned to the first loop. The minimum $g^{(2)}$ is 1.91 which corresponds to a Schmidt number $K = 1/(g^{(2)}_{\text{min}} - 1) \approx 1.1$. The dashed line at $g^{(2)} = 2$ corresponds to ideal single-mode squeezing ($K = 1$). Panel **b** shows the noise-reduction factor (NRF) vs. ϕ as measured using the first, second, and third loop, respectively. The minimum NRF values measured with each loop are 0.59, 0.61, and 0.64. The dashed line at NRF = 1 indicates the classical bound.

setup. These entangled pairs were created by coupling the first mode entirely into one loop delay and, after one circulation, interfering it with a second mode by setting the variable beam-splitter to 50 % transmittance. Imposed by the travel time in the delay lines, the entangled pairs are 1, 6 or 36 time bins (τ) apart, depending on which loop was used to generate them. This procedure is repeated for multiple pairs (54 pairs for the first loop, 72 pairs for second and third loops) with a different relative phase applied to each pair, allowing us to obtain the $g^{(2)}$ and noise-reduction factor for each loop as a function of the programmed phase ϕ (see Fig. S12).

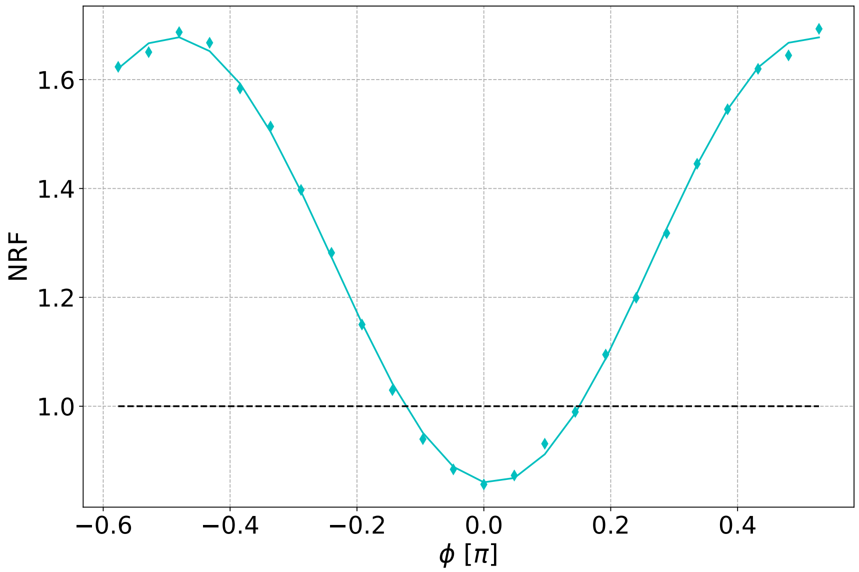

Figure S13. **Long-range two-mode squeezed states**. NRF vs. ϕ as measured using the third loop and a total of 5 round-trips leading to 180 time bins of separation between the modes analyzed. The minimum NRF value measured is 0.86. The dashed line at NRF = 1 indicates the classical bound.

To better address the question of how distinguishability [16] changes for larger pulse separations, we prepared and evaluated two-mode squeezed states using the third loop to delay one mode 5 times, resulting in interference between squeezed states emitted with 180 time bins of separation. The data for the NRF is shown in Fig. S13, and is fully consistent with our model of the device taking into account different noise channels, i.e. no significant increased distinguishability is observed within the limitations of our measurement.

3. UNITARY MATRIX

For our GBS instances, the arguments of the three phase-rotation gates were drawn from a uniform distribution bounded by $-\pi/2$ and $\pi/2$. The intensity transmittance of the three tunable beamsplitters can be programmed to take any values between 0 and 1. For example, in the $g^{(2)}$ and NRF scans (cf. Fig. S12) of each individual loop we bypass the other two loops by setting their respective beamsplitter transmissivity to zero. Similarly, the transmissivity is set to unity whenever we need to fill the loop delays with the initial squeezed-light modes and empty them at the end. Despite this high-degree of programmability, the family of unitaries accessible

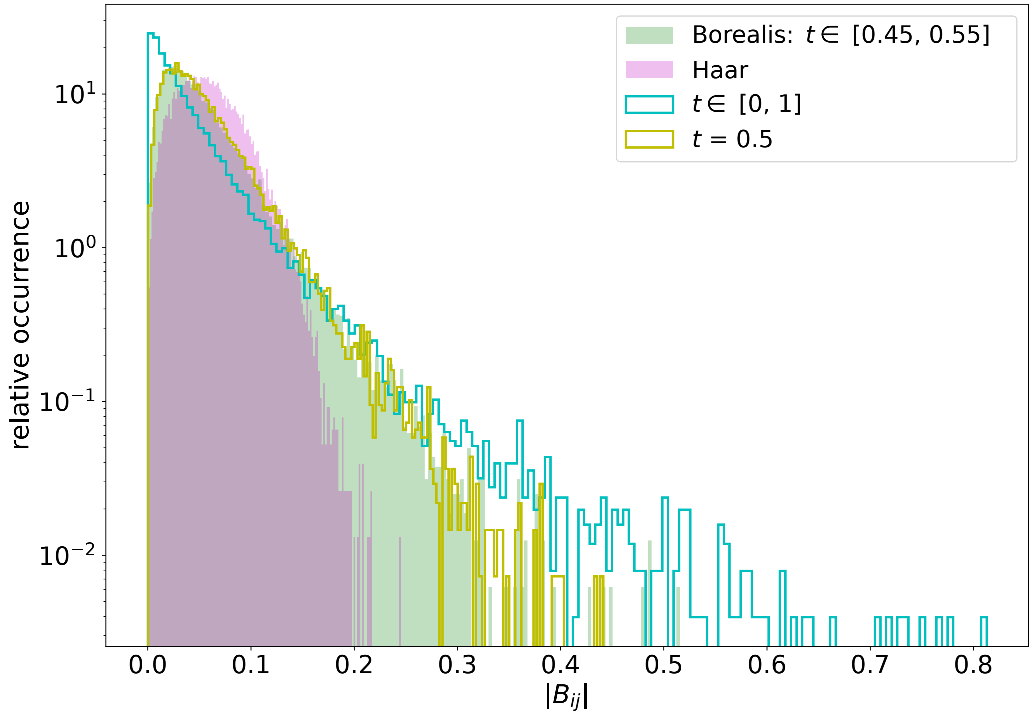

Figure S14. Distribution of elements of the adjacency matrix ($B = UU^T$) obtained from different families of random 216 × 216 unitaries. The green area illustrates the elements of a typical unitary applied by Borealis where the distribution of beamsplitter transmittances is bounded between 0.45 and 0.55. For comparison, the pink area corresponds to a Haar-random unitary. We observe that drawing from the full transmittance range (blue line) yields a sparser adjacency matrix, and that setting the beamsplitters to a static state of balanced transmission ($t = 0.5$, yellow line) will not approximate Haar-randomness better than a typical GBS instance executed by Borealis.

to our device does not cover densely the set of unitary matrices between all modes.

Moreover, for equally squeezed states interfering in a Haar-random unitary, and assuming negligible loss, the associated adjacency matrices of the Gaussian state would be given by $\boldsymbol{B} = \boldsymbol{U}\boldsymbol{U}^T$, and element of the circular orthogonal ensemble [4, 17]. Our choice of gate sequence was selected to ensure that the linear optical transformation used in our quantum advantage demonstration satisfied the requisite assumptions needed to exit the regime of classical simulability. Other gate sequences can be readily programmed into the device, but the regime of quantum advantage requires careful selection of gate settings within a specific range. Although our beamsplitters are able to apply any given transmittance between 0 and 1, we limit the interval of the distribution down to $t \in [0.45, 0.55]$, simply because the unitaries obtained $\boldsymbol{U}_{\text{loop}}$ yield a much denser adjacency matrix $\boldsymbol{B} = \boldsymbol{U}_{\text{loop}}\boldsymbol{U}_{\text{loop}}^T$. As Fig. S14 illustrates, biasing the beamsplitter transmittance distribution towards 50 % allows us to approximate matrices from the circular orthogonal ensemble much better compared to a scenario where the inten-

sity transmittance distribution is drawn uniformly between 0 and 1.

Note that the interferometer associated with a three-dimensional GBS instance leads to adjacency matrices with similar statistical properties to the ones of an interferometer drawn for the Haar measure. However, the losses associated with high-dimensional interferometer are much smaller. For the three dimensional instances where $M = a^3 (a = 6)$ modes go through loops accommodating 1, a and a^2, the dominant loss comes from the longest loop. If we associate η_{unit} as the loss from the shortest, one time-bin loop, then the largest loss comes from the longest loop and is given by $\eta_{net} = \eta_{unit}^{d^2} = \eta_{unit}^{M^{2/3}}$. If instead we had constructed a universal programmable interferometer where each mode sees M beamsplitters each with transmission η_{unit}, the net loss would accrue exponentially in the number of modes as $\eta_{net} = \eta_{unit}^{M}$ as elaborated in Ref. [17]

4. BENCHMARKING METHODS

4.1. Obtaining the transfer matrix

A quantum program is defined by some transfer matrix $\boldsymbol{T}$ (in our case of dimension 216 × 216) applied to squeezed input states, with squeezing array $\boldsymbol{r}$. Accurate knowledge of $\boldsymbol{T}$ is crucial to define the ground truth which our experimental data and all adversaries are compared against. Using the six arrays of gate arguments (three rotation gates, three beamsplitters) the phase offset associated with each loop and transmittance parameters, a user can conveniently obtain $\boldsymbol{T}$ using Xanadu's open-source photonic-simulation package StrawberryFields [18]. In particular, the set of parameters required to obtain a full description of the GBS circuit is

- `phi0`, `phi1`, `phi2` *(arrays)*: phase-gate arguments for each loop and time bin,
- `alpha0`, `alpha1`, `alpha2` *(arrays)*: beamsplitter arguments for each loop and time bin,
- `phi_loop0`, `phi_loop1`, `phi_loop2` *(floats)*: the phase offset associated with each loop,
- `eta_comm` *(float)*: the common transmittance to each mode
- `eta_loop0`, `eta_loop1`, `eta_loop2` *(floats)*: round-trip transmittances of the three loops
- `eta_ch_rel` *(array)*: the relative transmittance of the 16 demux/detector channels.

In order to not only obtain the transfer matrix $\boldsymbol{T}$ but also the ground truth, a user will also need knowledge of `r` *(array)*, representing the squeezing parameters of the 216 computational modes. Equipped with these parameters, $\boldsymbol{T}$ can be determined by the following code:

```
import numpy as np
import strawberryfields as sf
from strawberryfields.tdm.tdmprogram import get_mode_indices
from strawberryfields.ops import Sgate, Rgate, BSgate, LossChannel

modes = 216
delays = [1, 6, 36]
d = len(delays)
n, N = get_mode_indices(delays)

# loops are filled with 43 vacuum modes initially
vac_modes = sum(delays)
modes_tot = modes + vac_modes

# extend squeezing array by 43 zeros accounting for vacuum modes in the
    delay lines
r_tot = np.concatenate((r, (np.zeros(vac_modes))))

# phase offset in the loops
phi_loop = [phi_loop0, phi_loop1, phi_loop2]

# loop efficiencies
eta_loop = [eta_loop0, eta_loop1, eta_loop2]

# expand channel efficiencies to array of length modes_tot
reps = int(np.ceil(modes_tot / 16))
eta_ch_rel_tot = np.tile(eta_ch_rel, reps)[:modes_tot]
```

```python
# gate arguments for the sf.TDMProgram
gate_args = [r_tot, phi0, alpha0, phi1, alpha1, phi2, alpha2,
    eta_ch_rel_tot]

prog = sf.TDMProgram([N])
with prog.context(*gate_args) as (p, q):
    LossChannel(eta_comm) | q[n[0]]
    for i in range(d):
        Rgate(p[2 * i + 1]) | q[n[i]]
        BSgate(p[2 * i + 2], np.pi / 2) | (q[n[i]], q[n[i + 1]])
        Rgate(phi_loop[i]) | q[n[i]]
        LossChannel(eta_loop[i]) | q[n[i]]
    LossChannel(p[7]) | q[0]

prog.space_unroll(1)
prog = prog.compile(compiler="passive")

T_tot = prog.circuit[0].op.p[0]
T = T_tot[vac_modes:, vac_modes:]
```

In one straightforward step, we can add a squeezing operation to the above program which allows us to obtain the covariance matrix that represents the ground truth $(\boldsymbol{r}, \boldsymbol{T})$ of our GBS instances.

4.2. Photon-number statistics

The experimental data was benchmarked in different ways for the low, intermediate and high photon-number regime in the main text. In the low photon-number regime ($N < 10$) the number of possible outcomes is still small enough for us to sample the probabilities of each outcome experimentally and compare these probabilities against the predictions from the ground truth. In the intermediate photon-number regime ($N \sim 20$), the cross-entropy and

Bayesian benchmark are both based on evaluating the probability that a particular sample has been produced by the ground truth or one of the classical adversaries, respectively. In the high photon-number regime ($N > 30$, where computing these probabilities becomes intractable) we compare the statistical moments of the experimental samples against the ones of the different hypotheses. The next two paragraphs will sketch how we obtain sample probabilities and statistical moments that we used for performance benchmarking in the main text.

The probability to obtain a given sample under a certain transfer matrix $\boldsymbol{T}$ applied to a family of hypothetical optical input states was obtained in different ways: For indistinguishable (ground truth) and distinguishable squeezed states as well as for squashed and thermal states, we first built the quadrature-covariance matrix by applying $\boldsymbol{T}$ to the respective optical input states. The covariance matrix allowed us to obtain the adjacency matrix A from which we obtain the respective outcome probability by Equation (22). For coherent states with mean photon number $\langle n \rangle$, the probability to produce a sample $(n_1, n_2, \ldots, n_N)$ with total photon number N was obtained via $\exp(-N) \prod_i \langle n \rangle_i^{n_i} / \left(\prod_i n_i!\right)$. These methods to compute the probabilities of individual sample outcomes under different hypotheses were used for Figs. 2 and 3 in the main part. In particular, for Fig. 2 we computed these probabilities for all possible outcomes when N photons are to be placed in M time bins, grouped by the maximum amount of photons in one bin.

The photon-number distribution for the ground truth (squeezed states) and adversaries (squashed, thermal, and coherent states) used in Fig. 4a was obtained using the quadrature-covariance matrices of the respective hypotheses (note that the distribution of the distinguishable-squeezed adversary is by nature identical to the one of the ground truth and therefore not illustrated in the plot). From the quadrature-covariance matrices, we also derive the photon-number covariance matrices which describe correlations between modes i and j by $\mathrm{Cov}(n_i, n_j) = \langle n_i n_j \rangle - \langle n_i \rangle \langle n_j \rangle$. The elements of these photon-number covariance matrices (excluding $\mathrm{Cov}(n_i, n_i)$) are used for the scatter plots in Fig. 4b where they are compared against the experimental photon-number correlations.

5. SCALABILITY

The striking advantage of time-domain multiplexing is that the number of qumodes can be, in principle, increased with minimal effort, involving no additional hardware. The achievable

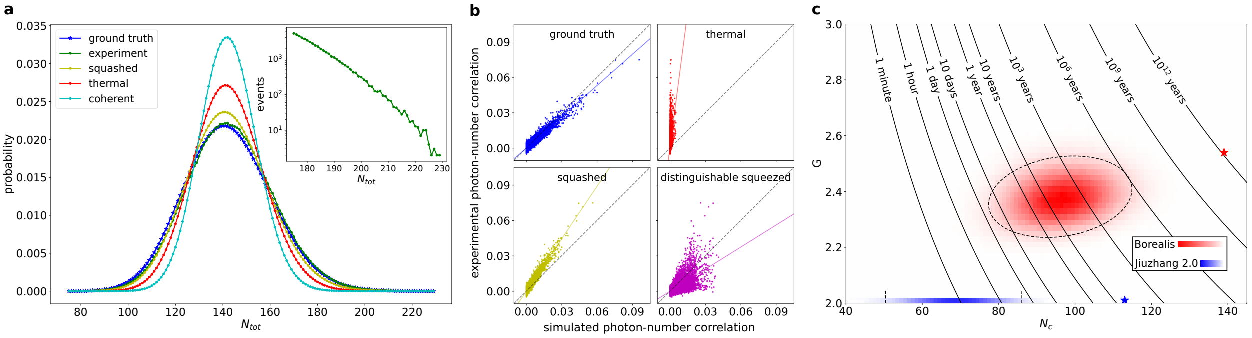

Figure S15. **Gaussian BosonSampling with 288 modes**. By simply increasing the number of squeezed-light pulses emitted by the source, for each sample containing M modes, we were able to sample GBS instances with 288 modes and a mean total photon number of 143.2. Similar to Fig 4 in the main text, panels **a** and **b** illustrate the photon-number distribution and second-order correlation of the experimental samples compared to the ones of the different hypotheses, and panel **c** shows the computation time required in order to simulate the experimental samples. The red star in panel **c** corresponds to our brightest sample containing 242 photons, while samples from Jiuzhang 2.0 (blue) are the same as for the main text.

number of modes in our experiment is currently limited by the repetition rate of the experiment. A 10 kHz clock provides a time window of 100 μs per shot (sample). After subtraction of the time it takes to relock and evacuate lock light and vacuum modes from the loops, we are left with 48 μs acquisition time which, at 6 MHz pulse rate, allows us to generate and measure up to 290 modes in each sample. Indeed, changing only a handful of lines in our code base allowed us to experimentally demonstrate GBS instances with 288 optical modes with a mean total photon number of 143.21 ± 0.02 and the brightest sample containing 242 photons, as illustrated in Fig S15. Decreasing the clock rate would immediately allow us to reach even higher number of modes. One should note, however, that increasing the acquisition time beyond what is sufficient for our demonstration, and thus disabling the locks for a longer time, may hinder the stability of the setup and possibly impose a different locking scheme.

NOTE ON COMMERCIAL EQUIPMENT

Certain commercial equipment, instruments, or materials are identified in this paper to foster understanding. Such identification does not imply recommendation or endorsement by the National Institute of Standards and Technology, nor does it imply that the materials or equip-

ment identified are necessarily the best available for the purpose.

[1] Barnett, S. & Radmore, P. M. *Methods in theoretical quantum optics*, vol. 15 (Oxford University Press, 2002).

[2] Barvinok, A. *Combinatorics and complexity of partition functions*, vol. 9 (Springer, 2016).

[3] Caianiello, E. R. On quantum field theory — i: explicit solution of dyson's equation in electrodynamics without use of feynman graphs. *Il Nuovo Cimento* **10**, 1634–1652 (1953).

[4] Hamilton, C. S. *et al.* Gaussian boson sampling. *Physical Review Letters* **119**, 170501 (2017).

[5] Bulmer, J. F. *et al.* The boundary for quantum advantage in gaussian boson sampling. *Science Advances* **8**, eabl9236 (2021).

[6] Quesada, N., Arrazola, J. M. & Killoran, N. Gaussian boson sampling using threshold detectors. *Physical Review A* **98**, 062322 (2018).

[7] Kaposi, Á., Kolarovszki, Z., Kozsik, T., Zimborás, Z. & Rakyta, P. Polynomial speedup in torontonian calculation by a scalable recursive algorithm. *arXiv preprint arXiv:2109.04528* (2021).

[8] Gupt, B., Izaac, J. & Quesada, N. The walrus: a library for the calculation of hafnians, hermite polynomials and gaussian boson sampling. *Journal of Open Source Software* **4**, 1705 (2019).

[9] Villalonga, B. *et al.* Efficient approximation of experimental Gaussian boson sampling. *arXiv preprint arXiv:2109.11525* (2021).

[10] Mehmet, M. *et al.* Squeezed light at 1550 nm with a quantum noise reduction of 12.3 db. *Optics Express* **19**, 25763–25772 (2011).

[11] Vaidya, V. D. *et al.* Broadband quadrature-squeezed vacuum and nonclassical photon number correlations from a nanophotonic device. *Science Advances* **6**, eaba9186 (2020).

[12] Takeda, S., Takase, K. & Furusawa, A. On-demand photonic entanglement synthesizer. *Science Advances* **5**, eaaw4530 (2019).

[13] Neuhaus, L. *et al.* Pyrpl (python red pitaya lockbox)-an open-source software package for fpga-controlled quantum optics experiments. In *European Quantum Electronics Conference*, EA_P_8 (Optical Society of America, 2017).

[14] Lita, A. E., Miller, A. J. & Nam, S. W. Counting near-infrared single-photons with 95% efficiency. *Optics Express* **16**, 3032–3040 (2008).

[15] Morais, L. A. *et al.* Precisely determining photon-number in real-time. *arXiv preprint arXiv:2012.10158* (2020).

[16] Shchesnovich, V. Distinguishability in quantum interference with the squeezed states. *arXiv preprint arXiv:2109.01857* (2021).

[17] Deshpande, A. *et al.* Quantum computational advantage via high-dimensional gaussian boson sampling. *Science Advances* **8**, eabi7894 (2022).

[18] Killoran, N. *et al.* Strawberry fields: A software platform for photonic quantum computing. *Quantum* **3**, 129 (2019).
